# Supercritical Water is not Hydrogen Bonded

**DOI:** 10.1002/anie.202009640

**Published:** 2020-09-04

**Authors:** Philipp Schienbein, Dominik Marx

**Affiliations:** ^1^ Lehrstuhl für Theoretische Chemie Ruhr-Universität Bochum 44780 Bochum Germany

**Keywords:** ab initio calculations, hydrogen bonds, molecular dynamics, supercritical fluids, water

## Abstract

Thinking about water is inextricably linked to hydrogen bonds, which are highly directional in character and determine the unique structure of water, in particular its tetrahedral H‐bond network. Here, we assess if this common connotation also holds for supercritical water. We employ extensive ab initio molecular dynamics simulations to systematically monitor the evolution of the H‐bond network mode of water from room temperature, where it is the hallmark of its fluctuating three‐dimensional network structure, to supercritical conditions. Our simulations reveal that the oscillation period required for H‐bond vibrations to occur exceeds the lifetime of H‐bonds in supercritical water by far. Instead, the corresponding low‐frequency intermolecular vibrations of water pairs as seen in supercritical water are found to be well represented by isotropic van‐der‐Waals interactions only. Based on these findings, we conclude that water in its supercritical phase is not a H‐bonded fluid.

## Graphical abstract

Ab initio molecular dynamics simulations are used to monitor the evolution of the H‐bond network of water from room temperature to supercritical conditions. The results reveal that the H‐bond oscillation period exceeds the lifetime of H‐bonds in supercritical water by far. Instead, the intermolecular vibrations of water pairs as seen in supercritical water are found to be well represented by isotropic van‐der‐Waals interactions only.

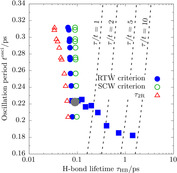

## Introduction

Hydrogen‐bonding and the resulting three‐dimensional network topology certainly is the hallmark of water^1^, ^2^, ^3^ and provides much of the mechanistic underpinnings of its many so‐called anomalies.^4^, ^5^ Thus, thinking about water implies thinking about H‐bonding. In this article, we are going to ask (and answer) the simple question if this remains true in the supercritical phase of water.

Recently, supercritical fluids and supercritical water (SCW) in particular have attracted enormous cross‐disciplinary attention^6^, ^7^, ^8^, ^9^ as “tunable solvent environments”^8^ in chemical synthesis^10^ and catalytic processes.^11^, ^12^ SCW is even envisaged as a mediator in nuclear power plants.^13^ Moreover, SCW and supercritical fluids in general, attracted interest in fundamental physics in view of putative transitions from liquid‐like to gas‐like regions discussed in terms of separating Widom, Frenkel or percolation lines.^14^, ^15^, ^16^, ^17^, ^18^, ^19^, ^20^ In nature SCW occurs in the earth mantle where it takes an active part in hydrothermal formation processes^21^ and it could be discovered close to so‐called “black smokers” at the bottom of the deep sea.^22^ In all these application and natural appearances SCW acts as a solvent. Solvation properties, at least in room temperature water (RTW), are inextricably linked to H‐bonds and the famous tetrahedral H‐bond network and therefore the question if H‐bonds exist in SCW and how their behavior changes with respect to RTW is of fundamental importance.

Given the importance of SCW in both, fundamental and applied science, its H‐bonding properties have been investigated since decades based on many complementary experimental approaches (examples given) such as neutron/X‐ray diffraction (ND/XRD),^23^, ^24^, ^25^ quasi‐elastic neutron scattering,^26^ deep inelastic neutron/X‐ray scattering,^27^ nuclear magnetic resonance (NMR)^28^, ^29^ or mid infrared (IR) as well as Raman spectroscopy;^30^, ^31^ recall that the critical point (CP) of water is located at *T*_c_=647 K, *p*_c_=221 bar, and *ρ*_c_=0.322 kg L^−1^ according to accurate experimental data.^32^ Computer simulations contributed a detailed molecular level picture of H‐bonding according to force field molecular dynamics (FFMD) simulations (e.g. refs. 15, 16, 17, 18, 20, 33, 34) and ab initio MD (AIMD) simulations^35^ (e.g. refs. 36, 37, 38).

The currently established picture concerning whether H‐bonds exist in SCW at all is primarily based on structural considerations in terms of ensemble averaged radial distribution functions (RDFs), in particular O‐O and O‐H RDFs. From ND experiments it was concluded that there is “*… little room for doubt that the hydrogen bond persists in the supercritical regime…”*,^39^ yet there is “*… a reduction of the H‐bond population in the supercritical state”*.^26^ Noteworthy, the H‐bonding feature of the O‐H RDF “*… has been washed out into a broad shoulder”* 40 in SCW while it is a prominent peak in RTW. This qualitative change of the RDF led to the question “*… as to whether this can still be regarded as hydrogen bonding at this temperature.”*.^40^ An alternative experimental approach is to analyze the time‐averaged proton NMR chemical shift as a function of density, temperature, and pressure. Here, it was concluded for SCW that “*… there are still 29 % as many hydrogen bonds at 400 °C and 400 bar (ρ*=0.52 g cm^−3^) *as for room temperature water”*.^28^ Note that these landmark papers from the mid 1990ies still represent the state‐of‐the‐art in the field even today.

Perhaps more importantly, it has been confirmed experimentally and computationally that the famous tetrahedral arrangement^41^ of the water molecules in RTW due to the preferred local fourfold coordination of the individual water molecules is completely lost in SCW.^24^, ^33^, ^38^, ^42^ Moreover, the extent of H‐bonding, as quantified by the number of H‐bonds per water molecule, systematically and significantly decreases as a function of increasing temperature.^33^, ^38^ Last but not least, the famous cooperative H‐bonding effect, which is responsible for the unusually large molecular dipole moment of H_2_O molecules in RTW,^43^ is drastically reduced in SCW.^38^ These experimental and computational findings indicate that H‐bonds are present in SCW, but are strongly weakened, and that the properties of the H‐bond network are significantly modified compared to RTW. This viewpoint culminates in the currently accepted picture of SCW as summarized in a recent authoritative monograph: “*There is sufficient evidence that hydrogen bonds do exist in SCW, with general agreement that the tetrahedral hydrogen‐bonded network present in ambient water is no longer present in SCW”*.^9^

Based on all these studies and clear statements it is nowadays broadly assumed at the outset that H‐bonds do exist in SCW without questioning it. This is despite the fact that reported orientationally and time‐averaged observables might also be explained differently than assuming sufficiently stable and properly directional water‐water arrangements (as we will demonstrate in what follows). This might also explain why there usually is no clear commitment made in recent studies if SCW is H‐bonded or not. In this vein, advanced experimental and computational studies tacitly assume the existence of H‐bonds when analyzing and interpreting the data and, thus, conclude that “H‐bonding is drastically reduced”, see for example, refs. 20, 27, 34, 38.

Very complementary to the aforementioned approaches is vibrational spectroscopy in the THz frequency window since that technique has been shown to most directly probe the H‐bond dynamics within the water network.^44^ Here, the famous H‐bond network mode is directly probed, which monitors the *intermolecular* hindered translations of the water molecules. THz spectroscopy is thereby different than both, traditional mid‐IR and Raman experiments where the H‐bond is only indirectly probed by induced changes of the *intramolecular* O‐H stretching motion, as well as from NMR, ND, or XRD experiments where a time‐averaged and mostly also orientationally averaged picture is obtained. In the case of RTW,^45^ the H‐bond network THz mode is located around 200 cm^−1^. It could be shown that this pronounced resonance is sensitive to local perturbations of the H‐bond network induced by for example, simple ions^46^, ^47^, ^48^, ^49^ or small molecules.^50^ Recently, the network mode has been shown to also respond very sensitively to increasing hydrostatic pressure.^51^ At supercritical conditions, preliminary FFMD simulations^18^ yielded qualitatively different THz spectra compared to RTW, however without being able to disclose the underlying molecular mechanism due to methodological shortcomings of the simulation method. Based on all this evidence accumulated in recent years only, it is therefore suggestive that the H‐bond THz mode should provide a most sensitive probe to also monitor H‐bonding in the supercritical state of water.

In this Research Article, we go back to square one and ask, in a fresh effort, the question if supercritical water is a H‐bonded fluid by using advanced simulation and spectral analyses techniques.

## Results and Discussion

### Ab Initio Supercritical Water

**Figure 1:**
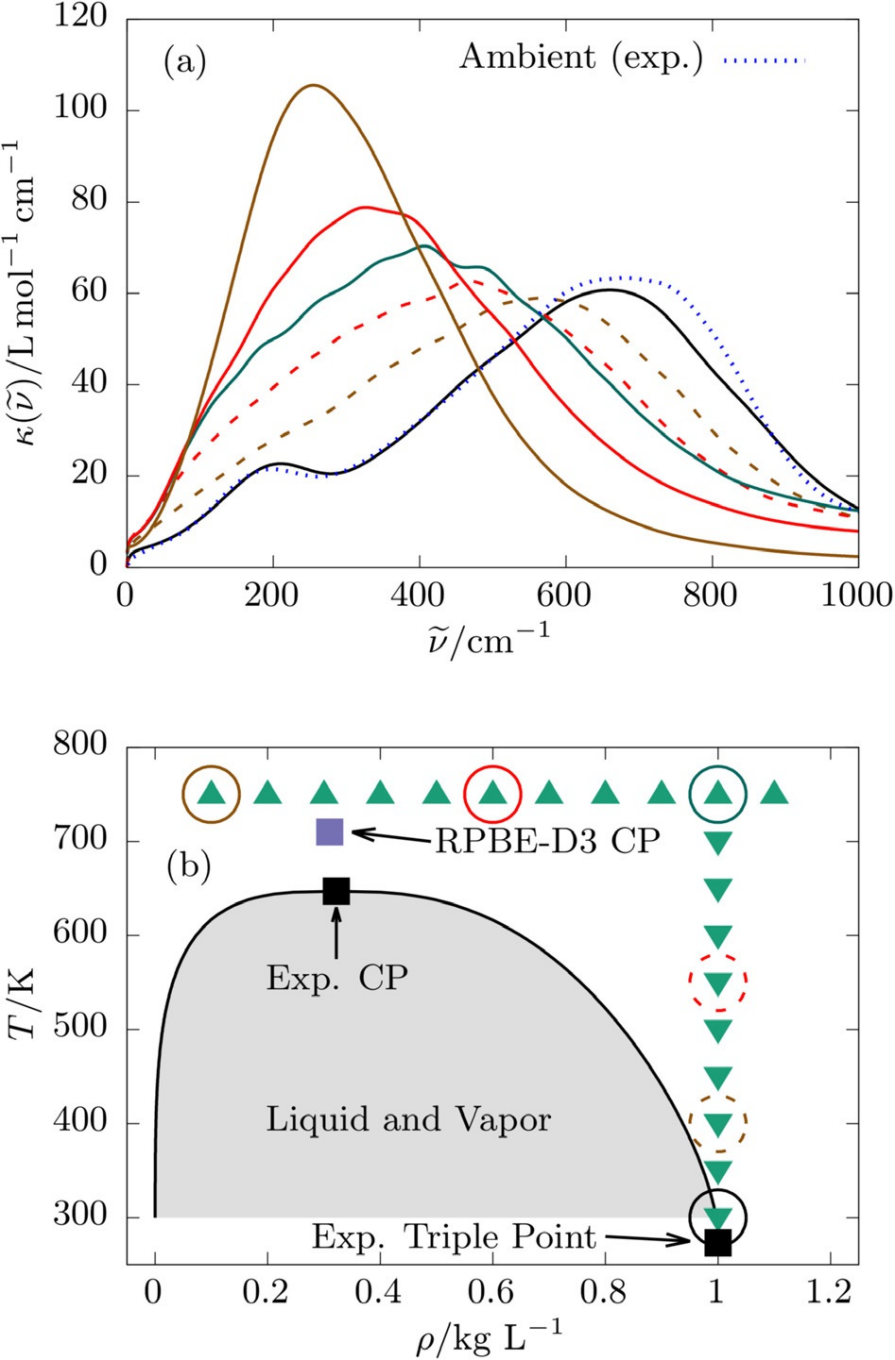
Molar THz absorption coefficients $\kappa \left(\tilde{\nu }\right)$ of room temperature and supercritical water (a) and the phase diagram of water as given by the accurate experimental IAPWS95 equation of state^32^ (b), where the coexistence curve is given as black solid line and the critical and triple points are marked using black solid squares. In panel (b), the green triangles mark all simulated state points on our isochore (down triangles) and isotherm (up triangles) scans; the violet square places the previously estimated CP of RPBE‐D3 water.^54^ Those state points where the THz spectra are presented in panel (a) are highlighted in (b) using colored circles and the very same color code as in (a). In panel (a), we present representative THz spectra computed from from RPBE‐D3 simulations of room temperature water (black solid line, RTW), of subcritical liquid water at 1.0 kg L^−1^ and temperatures of 400 and 550 K (brown and red dashed lines, respectively) as well as of supercritical water at 750 K and densities of 1.0, 0.6, and 0.1 kg L^−1^ (green, red and brown solid lines, respectively). The corresponding RTW experimental THz spectrum^45^ is reproduced in panel (a) as a blue dotted line for reference; note that neither the frequency nor the intensity of the computed THz spectra have been scaled or adjusted.

Our investigation is based on extensive AIMD simulations^35^ using the RPBE‐D3 functional which allows us to sample a total of more than 20 ns of AIMD trajectories using 128 water molecules; see SI for details. Only such long AIMD trajectories allow us to compute well‐converged THz spectra of SCW as illustrated in the SI. Concerning the choice of the functional, we note that RPBE‐D3 has been shown repeatedly by several groups to reliably represent both, RTW and SCW^38^, ^52^, ^53^, ^54^ with respect to experimental data. In AIMD, the computationally much more demanding revPBE0‐D3 hybrid functional has been demonstrated to provide an excellent representation of the full‐dimensional many‐body potential energy surface that describes RTW.^55^ In supporting Figure S1, we additionally compare the RDFs of SCW as obtained from RPBE‐D3 to the revPBE0‐D3 benchmark with most favorable agreement which explicitly validates the accuracy of RPBE‐D3 also for supercritical water. Finally, when it comes to H‐bond dynamics and THz spectroscopy, we refer to direct comparisons of our RPBE‐D3 results to NMR relaxation data (Figure 3 b) and to THz spectroscopy (Figure 1 a) with good agreement for these dynamical properties.

### THz Spectra and Two‐Body Vibrational Densities of States

We begin our discussion by presenting molar absorption coefficients, $\kappa \left(\tilde{\nu }\right)$ , in the THz regime (dubbed “THz spectra” for short) at selected super‐ and subcritical state points in Figure 1 (a). To illustrate the location of the shown state points we also present the corresponding phase diagram in Figure 1 (b). In case of RTW, our spectrum computed at 300 K agrees favorably with the experimental one^45^ and close to perfectly reproduces the absolute intensities and positions of the two prominent peaks around 200 and 650 cm^−1^ stemming from the intermolecular H‐bonding and the librational dynamics, respectively. The effect of thermal fluctuations on the THz spectrum is probed upon increasing the temperature until reaching supercritical conditions while keeping the density fixed at its RTW value, 1.0 kg L^−1^. The two distinct peaks are seen to vanish in favor of a single broad peak which, moreover, systematically red‐shifts as a function of increasing temperature. Once in the supercritical phase, decreasing the density of SCW isothermally at 750 K is found to systematically red‐shift that peak even more until it phenomenologically reaches at low density the frequency of the intermolecular H‐bonding peak of RTW, that is, roughly 200 cm^−1^.

**Figure 2:**
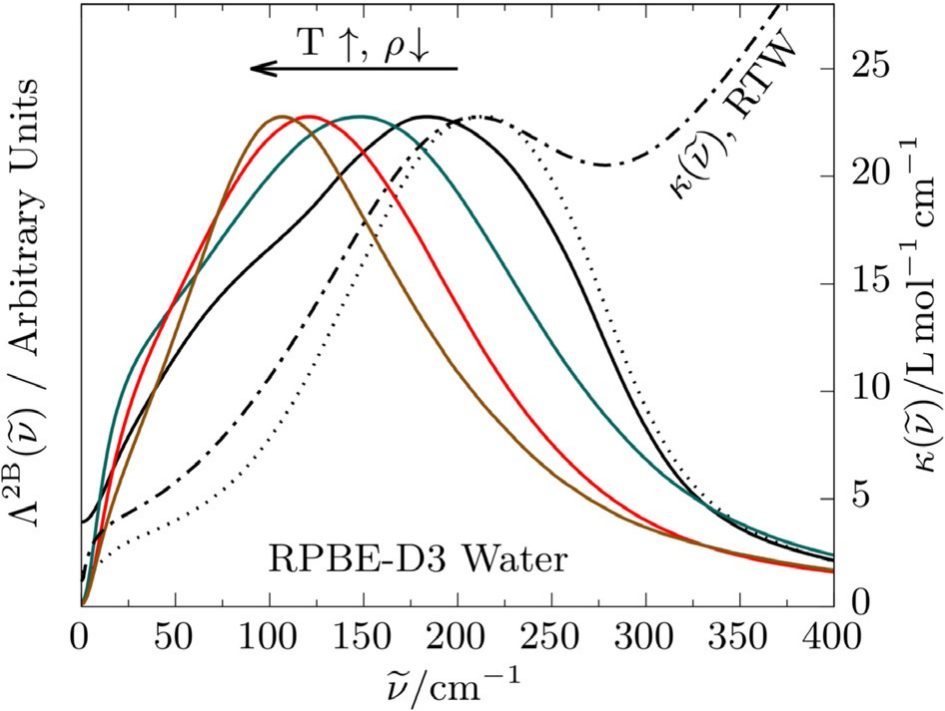
Two‐body spectral density [$\Lambda^{2B}\left(\tilde{\nu }\right)$ , Eq. (2)] from RPBE‐D3 simulations of RTW (black solid line) and SCW at 750 K and densities of 0.1 (brown line), 0.6 (red line), and 1.0 kg L^−1^ (green line). The black dotted line is $\Lambda^{2B}\left(\tilde{\nu }\right)$ obtained when applying a stricter OO cutoff criterion^57^ of 3.4 Å in case of RTW, see text, and the corresponding full THz spectrum of RTW ($\kappa \left(\tilde{\nu }\right)$ , right scale) is included as a black dashed‐dotted line for reference. Note that all $\Lambda^{2B}\left(\tilde{\nu }\right)$ spectra are scaled, see text, such that their maximum intensities are identical. The color code again corresponds to the highlighted state points in the phase diagram in Figure 1 (b).

Given these pronounced changes of the THz response, it is key to separate the H‐bond mode, being the prominent probe of H‐bonding dynamics in ambient liquid water,^44^, ^45^ from the librational band to assess their changes individually upon reaching supercritical conditions. In an effort to dissect the single broad peak in SCW in terms of molecular motion, we employ a projected relative velocity [Eq. (1)]^56^

(1) $$\Delta v_{IJ}\left(t\right)=\left({\vec{v}}_{I}\left(t\right)-{\vec{v}}_{J}\left(t\right)\right)\cdot {\vec{d}}_{IJ}\left(t\right),$$

where ${\vec{v}}_{I}\left(t\right)$ and ${\vec{v}}_{J}\left(t\right)$ are the center of mass velocities of two different water molecules and ${\vec{d}}_{IJ}\left(t\right)$ is the connecting vector between their centers of mass. The corresponding relative two‐body vibrational density of states (2B‐VDOS) is then given by [Eq. (2)]

(2) $$\Lambda^{2B}\left(\tilde{\nu }\right)\propto ℱ\left[\sum_{I<J}^{N}\left\langle \Delta v_{IJ}\left(0\right)\Delta v_{IJ}\left(t\right)\right\rangle \right],$$

where *N* is the total number of water pairs considered and $ℱ\left[\cdots \right]$ denotes the forward Fourier transform. We compute this specific spectral density for all water pairs whose centers of mass are closer than 4 Å, but irrespective if they are H‐bonded or not, meaning that their relative orientation is fully ignored. The resulting 2B‐VDOS is presented in Figure 2 at selected state points together with the computed THz spectrum of RTW as reference. Evidently, any vibrational DOS exclusively probes the particle dynamics and, thus, does not carry dipolar intensity (contrary to $\kappa \left(\tilde{\nu }\right)$ ), which allows us to scale their maxima to a convenient reference value for better comparison. Note that we have also separately determined the librational contribution $\Lambda^{\mathrm{rot}}\left(\tilde{\nu }\right)$ to the total THz band. It turns out that the THz spectrum of all SCW states is overwhelmingly dominated by this librational band, whereas $\Lambda^{2B}\left(\tilde{\nu }\right)$ contributes only little to the total THz response as detailed in the SI.

When directly comparing in Figure 2 the two‐body spectral density $\Lambda^{2B}\left(\tilde{\nu }\right)$ with the THz spectrum $\kappa \left(\tilde{\nu }\right)$ in the limit of RTW, one observes a slight red‐shift of the maximum and a shoulder around 75 cm^−1^. Both features can be tracked back to motion ranging beyond H‐bonded water pairs at RTW conditions because we employ a fairly large cutoff of 4 Å to still meaningfully identify water pairs in SCW (where the average OO distances are considerably longer in particular at low densities). Importantly, when reducing that cutoff to a value of 3.4 Å as appropriate for RTW,^57^ $\Lambda^{2B}\left(\tilde{\nu }\right)$ exactly reproduces the THz response $\kappa \left(\tilde{\nu }\right)$ of the H‐bond spectral feature of RTW, see dotted line in Figure 2. Therefore, $\Lambda^{2B}\left(\tilde{\nu }\right)$ is indeed capable to perfectly represent the intermolecular H‐bond stretching motion of RTW (giving rise to its THz network peak) and, thus, does monitor the intermolecular stretching motion of water pairs in more general terms, that is, without taking the relative orientations into account. Now, increasing the temperature at constant density, 1.0 kg L^−1^, the pronounced $\Lambda^{2B}\left(\tilde{\nu }\right)$ peak is found to systematically red‐shift from a maximum frequency of 180 cm^−1^ in case of RTW to about 150 cm^−1^ at 750 K in SCW according to Figure 2. Decreasing next the density at that supercritical temperature, the maximum frequency is seen to red‐shift even further down to about 100 cm^−1^ at 0.1 kg L^−1^.

At this stage, it seems that the analyses presented so far strongly support the notion that SCW remains H‐bonded even in the low‐density limit. The only difference of supercritical compared to ambient water appears to be a pronounced red‐shift of the H‐bond resonance from 200 down to 100 cm^−1^, see Figure 2, which in turn gets simply masked by the much more intense low‐frequency wing of the pronounced librational band since that shifts dramatically from ≈650 at RTW to ≈250 cm^−1^ in low‐density SCW, see Figure 1. It will be demonstrated in what follows that this suggestive conclusion does not hold true.

### Hydrogen‐Bond Lifetimes and Reorientational Relaxation Times

As the next step we analyze the reorientational and H‐bond dynamics in terms of the associated relaxation and lifetimes,^58^ respectively, when moving from RTW along subcritical states to the supercritical phase of water. These dynamical properties complement the analyses of intermolecular H‐bond vibrations and have been proven valuable to investigate SCW by FFMD and AIMD simulations, see for example, refs. 19, 38, 56, 59. Taking into account the well‐known ambiguities in the selected H‐bond criterion,^19^, ^20^, ^33^, ^38^ existing studies nevertheless broadly agree that the continuous H‐bond lifetime *τ*_HB_ is more than one order of magnitude smaller compared to RTW^9^, ^38^, ^56^, ^59^, ^60^ and amounts to about 100 fs in SCW including long‐time tails.

In order to provide a physical observable that on the one hand probes the dynamics within the water network, but on the other hand is independent on any H‐bond criterion and thus H‐bonding bias, we analyze now the reorientational relaxation time [Eq. (3)]^29^

(3) $$\tau_{2R}=\int_{0}^{\infty }\left\langle {\frac{3}{2}\cos}^{2}\Theta \left(t\right)-\frac{1}{2}\right\rangle dt,$$

where Θ(*t*) is the angle between the unit vector of the intramolecular O−H bond at time *t* and at time 0. Being a proper observable, *τ*_2R_ can not only be computed but also determined experimentally by NMR relaxometry even in SCW.^29^ Obviously, *τ*_2R_ is a single‐molecule quantity and, therefore, cannot be directly compared to H‐bond lifetimes, but both quantities are certainly closely related: At high densities, the average coordination number (within a distance radius of 3.43 Å) per water molecule, *n*_c_, is rather large in SCW, for example, *n*_c_>3 for densities exceeding 0.6 kg L^−1^ and *n*_c_≈5 at 1.0 kg L^−1^ for RPBE‐D3 water.^38^ If a molecule rotates in such an environment at least one H‐bond must be broken,^61^ irrespective of the rotation axis, and it follows that H‐bond and reorientational dynamics must be closely related at the level of their intrinsic time scales. Therefore, *τ*_2R_ provides an independent, measurable observable to probe H‐bond motion that is completely unrelated to any H‐bond definition. It thus does not suffer from the well known dispersion of H‐bond lifetimes reported in the literature.

**Figure 3:**
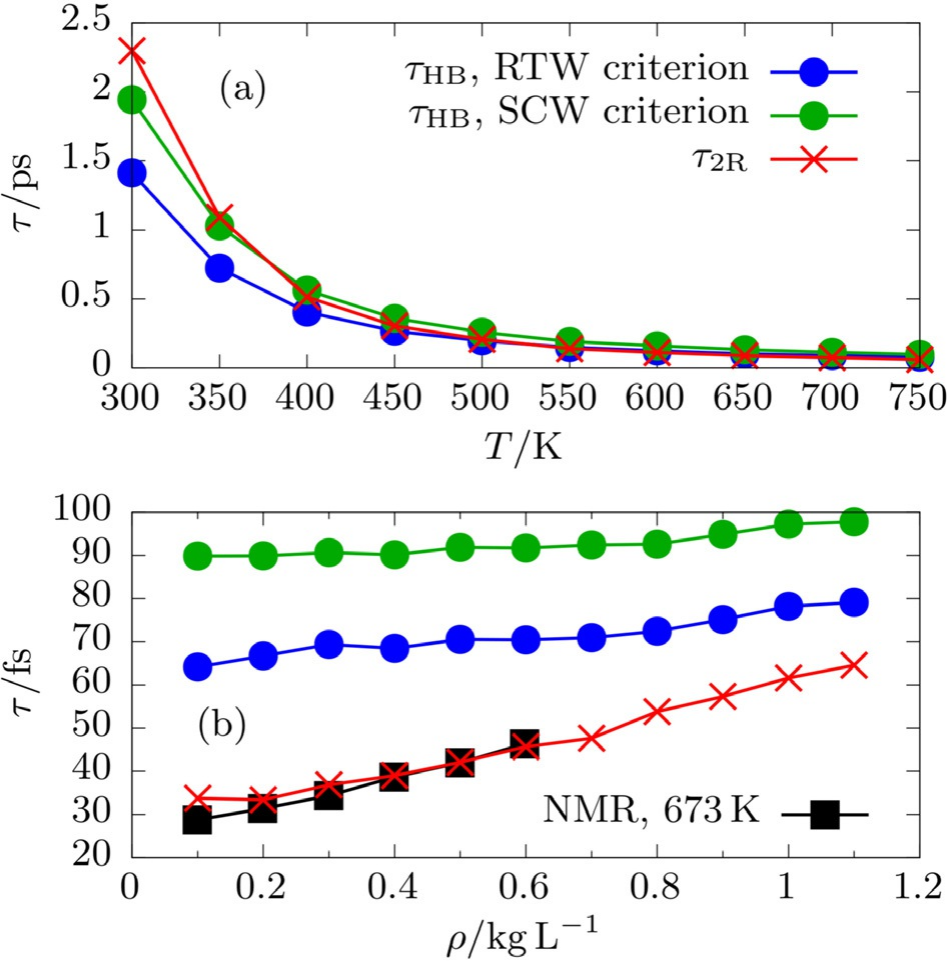
(a) Continuous H‐bond lifetimes, *τ*_HB_, from RPBE‐D3 simulations based on two distinct H‐bond criteria (RTW: blue circles, SCW: green circles) together with the reorientational relaxation time, *τ*_2R_, according to Equation (3) (red crosses) along the investigated isochore at 1.0 kg L^−1^ from RTW towards a supercritical temperature of 750 K. (b) Same properties as in (a) using the same color code but along the supercritical isotherm at 750 K as a function of density from 0.1 to 1.1 kg L^−1^. Experimental NMR *τ*_2R_ data^29^ at a comparable reduced temperature of T*=*T*/*T*_c_=1.040 are shown as reference (black squares) for the RPBE‐D3 data (red crosses) without any adjustments; recall that our simulations are conducted at T*=1.056. The H‐bond lifetimes are adapted from ref. 38.

Turning now to the data in Figure 3 (a), both time scales, *τ*_HB_ and *τ*_2R_, are found to dramatically decrease with respect to RTW when the temperature is increased. This qualitative behavior is not unexpected since the H‐bond lifetime follows an Arrhenius‐type behavior^62^ as indeed explicitly confirmed by us for RPBE‐D3 water.^38^ Given that *τ*_HB_ is sensitive to the H‐bond criterion we used our RTW and SCW criteria throughout as explained in the SI. Interestingly, the impact of these two quite different criteria is seen to be negligible on the scale of the physical changes of that lifetime as a function of temperature. Turning now to the supercritical isotherm at 750 K in panel (b), the reorientational relaxation time *τ*_2R_ is found to systematically and significantly increase with increasing density, from about 34 fs at 0.1 kg L^−1^ to 65 fs at 1.1. kg L^−1^ Importantly, our RPBE‐D3 values of *τ*_2R_ perfectly match the available experimental NMR data^29^ at a comparable temperature of 673 K (corresponding to T*=1.040 given that our simulations are conducted at T*=1.056).

Overall, numerous computational studies which use vastly different H‐bond criteria, sampling protocols and water models predict H‐bond lifetimes of about 100 fs in SCW.^9^, ^38^, ^56^, ^59^, ^60^ Moreover, our computed reorientational relaxation times, which do not depend on any H‐bond definition, excellently agree with available experiments and yield values somewhat smaller than 100 fs. Even a gross extrapolation of the H‐bond lifetime from RTW utilizing the Arrhenius‐type behavior^62^ (which is observed irrespective of the given H‐bond criterion^38^) yields a H‐bond lifetime of 87 fs in SCW, and thus is consistent with both, the literature and our study. We are therefore confident that H‐bond lifetimes not exceeding 100 fs in SCW as computed by us are reasonable estimates of the true dynamical behavior of SCW as quantified in terms of the lifetimes of intermolecular H‐bonds.

### Lifetimes vs. Intermolecular Vibrational Periods

**Figure 4:**
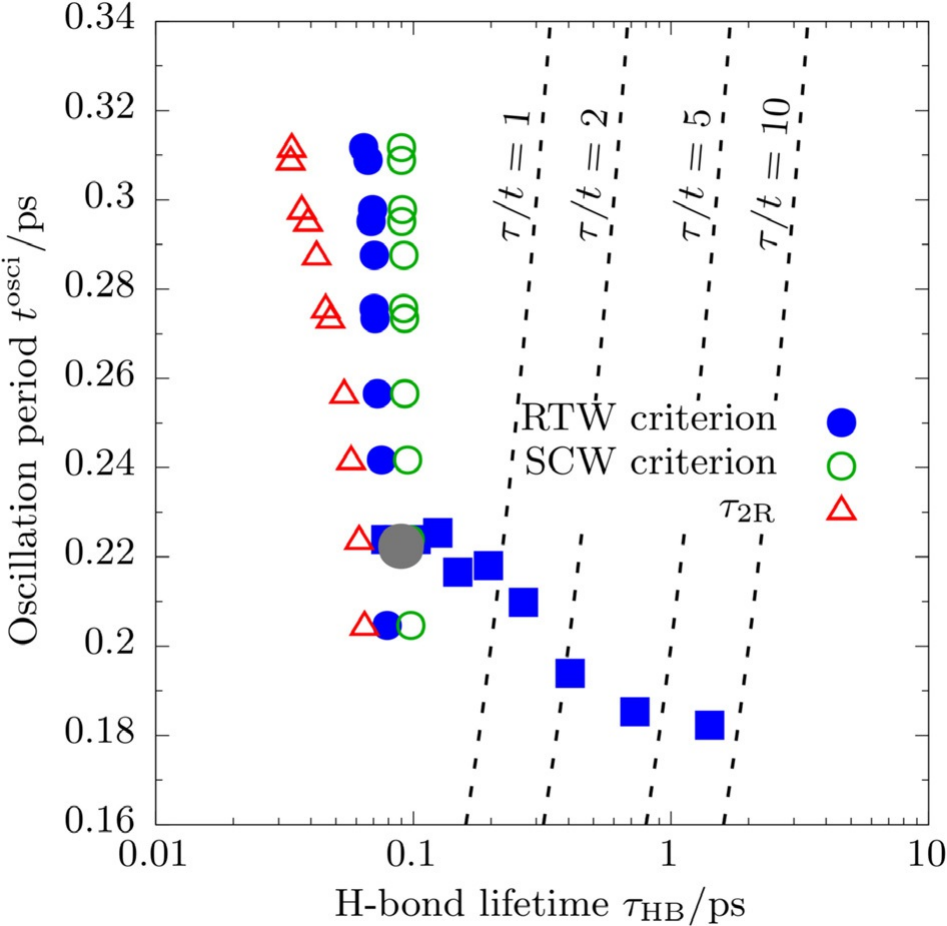
Oscillation period of the intermolecular stretching mode, *t* ^osci^ plotted against the continuous H‐bond lifetime, *τ*_HB_; see text for definitions and note the logarithmic scale of the abscissa. The dashed lines mark the regime to the left where the H‐bond lifetime is smaller than a certain multiple *k* of the oscillation period, *τ*_HB_=*n*⋅*t* ^osci^ where *n=*1,2,5,10. The H‐bond lifetimes are determined using the RTW and SCW criteria (blue and green, respectively) for all supercritical state points (circles) to demonstrate their invariance w.r.t. the definition, while the RTW criterion is applied to all subcritical states (filled squares) for simplicity. The subcritical state point of highest temperature, 700 K (i.e. T*=0.93), is marked using a large grey circle. In addition, the reorientational relaxation time, *τ*_2R_, along the supercritical isotherm is shown using red triangles.

Having now quantitative access to these molecular time scales, we can compare them in Figure 4 to the oscillation periods of the intermolecular stretching vibrations *t* ^osci^ (as quantified by the maxima of the corresponding two‐body spectral densities $\Lambda^{2B}\left(\tilde{\nu }\right)$ shown in Figure 2). In case of RTW, we find an oscillation period of about 0.18 ps and a H‐bond lifetime of approximately 1.41 ps. This implies that H‐bonds oscillate roughly ten times before they break in RTW. This familiar picture of the H‐bond water network changes dramatically when increasing the temperature towards supercritical conditions. At 750 K and at the same density as RTW, 1.0 kg L^−1^, we find *t* ^osci^≈224 fs whereas *τ*_HB_ is even less than half of it, only about 78 fs. Note that from a spectroscopic viewpoint this corresponds to values of about 150 and 430 cm^−1^, respectively, and thus corresponds to very distinct frequency regimes in vibrational spectroscopy!

In other words: The lifetime of putative H‐bonds is much shorter than the oscillation period of the intermolecular stretching vibrations due to hindered translations! This observation holds true for all investigated state points of supercritical water, irrespective of their density and irrespective of the chosen H‐bond criterion, and is confirmed when using the experimentally accessible reorientational relaxation times instead, *τ*_2R_.

The fact that the H‐bond lifetime is much smaller than the oscillation period of the intermolecular vibrations imposes important consequences for H‐bonding in SCW. It implies that H‐bonds are broken while an intermolecular vibration is still ongoing. Hence, these short‐range hindered translations are unaffected by any orientational directionality. This utmost dynamical picture of intermolecular encounters in SCW stimulates the question if it is meaningful to call SCW “H‐bonded” if on average a H‐bond does not even survive a single intermolecular oscillation period.

How does our conclusion of supercritical water not being H‐bonded compare to existing viewpoints regarding its H‐bonding properties? In a nutshell, THz spectroscopy offers a direct and, most importantly, time dependent approach to study intermolecular vibrations in water. In contrast, ND/XRD and NMR spectroscopy only yield a time‐averaged picture. Our conclusion does not imply that there are no structural H‐bond motives at all, however, they are remarkably short‐lived. This means that all H‐bond contacts are counted by such time‐averaged methods although they exist only fleetingly. In Sec. IV in the SI we more elaborately discuss that our conclusion as concisely announced by the title of this publication is indeed perfectly consistent with existing experimental data.

Mid‐IR spectroscopy, differently from diffraction, probes H‐bonds by using the *intramolecular* O‐H stretching dynamics as a proxy.^63^ These vibrations are located around 3500 cm^−1^ and are thus more than one order of magnitude faster than the *intermolecular* vibrations probed at THz frequencies. It follows that the *intramolecular* O‐H stretching vibrations are fast enough to detect also very short‐lived instantaneous H‐bond contacts, although these contacts exist too shortly to be recognized by the much slower *intermolecular* O⋅⋅⋅O stretching vibrations detected by THz radiation. Indeed, we show in supporting Figure S6 that the very pronounced red‐shift of the O‐H stretch in RTW, being the hallmark of H‐bonding in liquid water (where intermolecular O⋅⋅⋅H distances are short and O−H⋅⋅⋅O angles close to linear), gets dramatically reduced in SCW. Note that the same observation as obtained here from AIMD was also made recently in a sophisticated quantum‐classical study of the intramolecular stretching vibrations of SCW.^34^ At the level of the underlying structural dynamics^64^, ^65^, ^66^, ^67^ such surprisingly small O‐H shifts in SCW directly correlate with an enormously enhanced population of distinctly non‐linear intermolecular O−H⋅⋅⋅O orientations, which red‐shift much less even if the two water molecules come close. Here, this enhanced population of non‐linear orientations in SCW is statistically captured by the joint distance‐angle distribution functions in supporting Figure S7: Whereas in RTW the majority of nearest‐neighbor water‐water orientations is quasi‐linear as expected for H‐bonded liquids, the majority of them is indeed distinctly non‐linear in SCW even at densities that exceed that of RTW (see Sec. II.D in the SI for details). This essentially flat distribution of water‐water orientations, where quasi‐linear O−H⋅⋅⋅O H‐bonding arrangements are scarce compared to strongly bent orientations, is due to the dramatically decreased reorientational relaxation time in SCW (from roughly 2500 fs in RTW to ≈60 fs in SCW at 1.0 kg L^−1^ according to Figure 3 b), which makes proper H‐bonding orientations ultra‐short lived and thus transient in SCW. Remarkably, even in case of the highest density SCW this flat distribution of water‐water orientations is observed, although the coordination number exceeds six. In other words: Such orientations, which look like H‐bonds on an ultra‐short timescale, are mechanistically due to essentially isotropic statistical encounters of two water molecules at high temperatures, rather than due to directional intermolecular bonding. We are going to provide strong evidence in the following section that this picture indeed holds true.

### Coda: Supercritical Water as an Isotropic van‐der‐Waals Fluid

What else can the physical underpinnings be that lead to signatures in observables that have long been considered to support H‐bonding in SCW? To answer that question, we go back to the peak of the two‐body spectral density $\Lambda^{2B}\left(\tilde{\nu }\right)$ analyzed in Figure 2 with the aim to understand to which kind of vibrational motion this outstandingly pronounced resonance corresponds to in SCW. At ambient conditions, we have already demonstrated that it unambiguously corresponds to intermolecular H‐bond stretching vibrations along essentially linear donor‐acceptor arrangements within the tetrahedral H‐bond network, thus confirming H‐bonding in RTW. However, we have also shown that at supercritical conditions, the H‐bond lifetime is way too short to support the same interpretation.

As a first step toward understanding, we probe the role that directional H‐bonding plays for the hindered translational dynamics in SCW by using a standard water model that is well‐suited to describe SCW,^18^ namely SPC. However, in order to probe the impact of H‐bonding on the structural dynamics of water, we have switched off all those directional intermolecular water‐water interactions which imprint the respective orientational dependences (as described in more detail in the SI), thus leaving us with the corresponding purely isotropic Lennard‐Jones interactions between the oxygen sites only. In the absence of any directional H‐bonding, this so‐called LJ‐wat model enables us to qualitatively disentangle the spectral response of SCW at THz frequencies due to directional H‐bonding from that due to purely isotropic van der Waals bonding.

In practice, we use these simple LJ‐wat reference simulations exclusively to systematically analyze the low‐frequency vibrations when moving from RTW to hot subcritical to supercritical conditions as a function of temperature and density—but without any H‐bonds being present. Therefore, no short‐range tetrahedral orientational order is imprinted at all. Let us note in this context that Rahman^68^ already realized that there are indeed low‐frequency vibrations (unveiled by him using the standard single‐particle vibrational DOS) between the individual particles in the subcritical LJ liquid which lead to a pronounced resonance exclusively due to hindered translational motion (obviously in the absence of any angular interactions and thus orientational order). He has also worked out that this dynamical resonance of the subcritical LJ liquid is distinctly different from that of a Langevin liquid which is only subject to ballistic motion but not to any van der Waals attraction as the LJ fluid. Coming now back to SCW, in order to compare LJ water to realistic water, it must be considered that the critical points of LJ‐wat and RPBE‐D3 water differ quantitatively. For the sake of mapping, we therefore use the principle of corresponding states to obtain comparable state points; we refer to the SI for details as well as for as comparison of the LJ‐wat and RPBE‐D3 phase diagrams. In full analogy to our RPBE‐D3 simulations, we sampled the LJ‐wat fluid along a corresponding supercritical isotherm from very low to very high densities at the corresponding reduced temperature T*=*T*/*T*_c_=1.056 as well as along a corresponding isochore from the triple point up to that supercritical isotherm as illustrated in supporting Figure S2.

**Figure 5:**
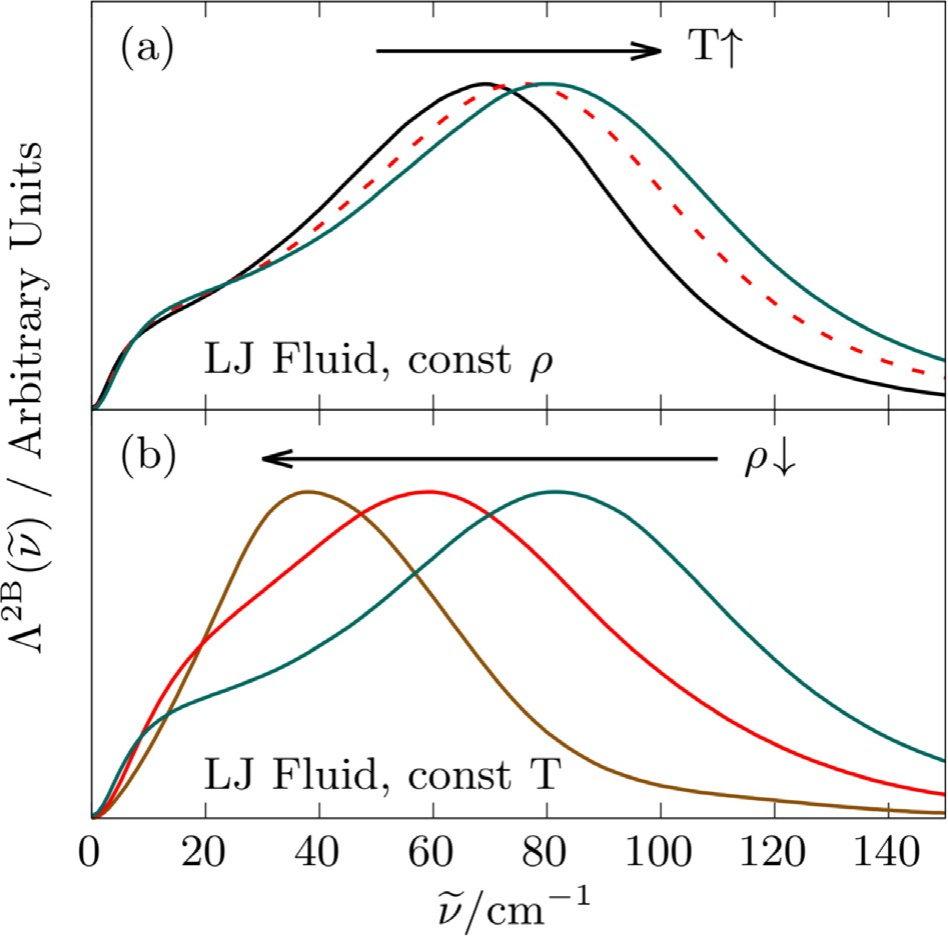
(a) Two‐body spectral density $\Lambda^{2B}\left(\tilde{\nu }\right)$ of the LJ‐wat fluid, see text, at selected temperatures along an isochore from its triple point up to the supercritical isotherm in (b). (b) Two‐body spectral density $\Lambda^{2B}\left(\tilde{\nu }\right)$ of the LJ‐wat fluid at selected densities along the supercritical isotherm at a comparable reduced temperature (T*=1.056) according to the principle of corresponding states. Selected thermodynamic states in (a) and (b) corresponding to Figure 2 and are depicted using the same line style and color to compare LJ‐wat to RPBE‐D3 water. Note that all $\Lambda^{2B}\left(\tilde{\nu }\right)$ spectra are scaled, see text, such that their maximum intensities are identical. The color code again corresponds to the highlighted state points in the phase diagram in Figure 1 (b). In the SI we detail how exactly thermodynamic state points of the RPBE‐D3 model and a simple LJ fluid model can be compared.

The resulting two‐body spectral density $\Lambda^{2B}\left(\tilde{\nu }\right)$ of the LJ‐wat fluid along the isotherm and isochore scans are depicted in panels (a) and (b) of Figure 5, respectively, in direct comparison to those of RPBE‐D3 water in Figure 2 using the same line code. In the supercritical phase, see Figure 5 (a), a systematic red‐shift of the hindered translational mode of the simple LJ‐wat fluid is observed when the density is decreased along the supercritical isotherm. This implies that, indeed, LJ‐wat reproduces at supercritical conditions the same qualitative trend as observed for SCW in Figure 2, but obviously without any directional order (meaning here H‐bonding) being present. The situation is distinctly different, however, when starting from ambient conditions, corresponding to RTW, and heating the LJ‐wat liquid up to the supercritical isotherm (while keeping the RTW density constant) as compiled in Figure 5 (b). Now, the hindered translational LJ‐wat mode blue‐shifts as a function of increasing temperature, while RPBE‐D3 water shows exactly the opposite qualitative behavior, that is, the H‐bond mode in Figure 2 red‐shifts as a function of increasing temperature from RTW to SCW. Given these facts, one must conclude that preferred water‐water orientations are key to describe the low‐frequency intermolecular motion in RTW, being a H‐bonded liquid, while they are not at all required to describe that same resonance in SCW.

How can this qualitative difference in the super‐ and subcritical phases of water be interpreted? There are evidently no directional (i.e. H‐bonding) interactions whatsoever operational in the LJ‐wat fluid, being exclusively subject to isotropic van der Waals interactions, which perfectly describe simple liquids such as rare gas atoms. This implies that any low‐frequency resonance observed in the LJ‐wat fluid must be unrelated to any tetrahedral directional H‐bond dynamics but rather exclusively due to isotropic interactions. Yet, the spectral changes of that intermolecular resonance $\Lambda^{2B}\left(\tilde{\nu }\right)$ in the supercritical phase of RPBE‐D3 water in response to changing the density are perfectly captured by the LJ‐wat fluid. The LJ‐wat fluid, however, qualitatively fails to describe the corresponding spectral changes observed when isochorically cooling the supercritical phase until ambient temperature is reached where H‐bonding interactions are decisive to describe RTW. The most obvious (Occam's Razor type) inference based on these facts is that tetrahedral directionality and thus H‐bonding do not play any role in the supercritical state of water, whereas they clearly do in subcritical water.

## Conclusions and Outlook

In conclusion, we unveil that the H‐bond lifetime in supercritical water is on average shorter than a single oscillation period of an intermolecular vibration between two adjacent water molecules. This rises the question if supercritical water should be considered as “H‐bonded”. On the one hand, our ab initio simulation results are shown to nicely agree with long existing experimental data in the supercritical phase of water, such as reorientational relaxation times obtained from NMR relaxometry or orientationally and time‐averaged radial distribution functions from XRD or ND experiments. On the other hand, our original time‐dependent and orientation‐resolved analyses of the structural dynamics and, in particular, the low‐frequency vibrational spectral response do not support the notion that supercritical water is a H‐bonded fluid.

Instead, we rather show that the low‐frequency intermolecular vibrations—that are clearly detected in supercritical fluid water at THz frequencies—are unambiguously due to isotropic water‐water contacts, which of course include ultrashort‐lived linear donor‐acceptor arrangements among many others orientations. This scenario is in stark contrast to ambient liquid water where the THz resonance is very clearly ascribed to long‐lived linear donor‐acceptor arrangements and thus to the tetrahedral H‐bonded water network. Here, long‐lived implies that many water‐water oscillations are possible in linear donor‐acceptor arrangements being the hallmark of H‐bonding, whereas short‐lived means that not even a single such intermolecular oscillation is possible. As such, the underlying hindered translational motion of water molecules at supercritical conditions does not correspond to intermolecular H‐bond stretching vibrations in highly directional tetrahedral arrangements, as opposed to ambient liquid water. This is the reason why the hindered translational motion, and thus the low‐frequency vibrational spectral response, in supercritical water is same as that of supercritical van der Waals fluids. The latter are clearly not subject to any directional H‐bonding and, thus, can be perfectly described using the purely isotropic Lennard‐Jones interactions as we explicitly demonstrate here for the supercritical state. We think that the absence of H‐bonding is the fundamental reason why supercritical water is a distinctly different solvent than ambient liquid water.

## Conflict of interest

The authors declare no conflict of interest.

## Supporting information

As a service to our authors and readers, this journal provides supporting information supplied by the authors. Such materials are peer reviewed and may be re‐organized for online delivery, but are not copy‐edited or typeset. Technical support issues arising from supporting information (other than missing files) should be addressed to the authors.

Supplementary
