# Supplementary material for: Supercritical Water is not Hydrogen Bonded
Source: Angew Chem Int Ed Engl. 2020 Sep 4;59(42):18578–85. doi: 10.1002/anie.202009640 (PMC7589343; doi:10.1002/anie.202009640)
Supplement: Supplementary file 1 — Supplementary [file ANIE-59-18578-s001.pdf]

Angewandte Chemie
Eine Zeitschrift der Gesellschaft Deutscher Chemiker

Supporting Information

Supercritical Water is not Hydrogen Bonded

Philipp Schienbein and Dominik Marx**

anie_202009640_sm_miscellaneous_information.pdf

CONTENTS

I. Simulations 2
A. Ab Initio Supercritical Water 2
B. Computational Details 3
C. Assessing the Density Functional: RPBE-D3 versus revPBE0-D3 4
D. Van der Waals Reference Simulations: Corresponding States of RPBE-D3 Water and the Lennard-Jones Fluid 6

II. Spectral Analyses 8
A. Computing Linear Absorption Coefficients 8
B. Hindered Translations: Two-Body Vibrational Density of States 10
C. Hindered Rotations: Librational Density of States 13
D. Mid-IR Spectroscopy: Intramolecular O-H Stretch 16

III. Structural Analyses 22
A. Joint Distribution Functions 22
B. Hydrogen-Bond Criteria 24

IV. Detailed Comparison between our Conclusions and Available Literature Viewpoints 24

References 28

I. SIMULATIONS

A. Ab Initio Supercritical Water

Our investigation is based on extensive AIMD simulations [1] using the RPBE-D3 functional which allows us to sample a total of more than 20 ns of AIMD trajectories using 128 water molecules. These long AIMD trajectories allow us to compute well-converged THz spectra of SCW as illustrated in Fig. S3 below. Moreover, it is well known that the correlation time of the density fluctuations diverges at the CP and that remnants reach into the supercritical phase of water [2]. Although we are not simulating directly at the CP, this certainly implies that the simulations need to be long enough to ensure sufficient sampling, which is why we developed a special protocol to extensively sample SCW even when using AIMD [3, 4]. Next, these electronic-structure-based AIMD simulations also include the intermolecular electronic polarization and charge transfer effects, which are required to correctly reproduce the experimental THz spectrum of RTW [5, 6] and to also describe the decisively smaller effective molecular dipole moment in SCW compared to RTW [4, 7].

Concerning the choice of the functional, we note that RPBE-D3 has been shown repeatedly by several groups to reliably represent many experimental properties of aqueous systems [8–13] including those of water far from ambient conditions at multi-kbar pressures [11, 14, 15]. Moreover, we could show recently that RPBE-D3 water reproduces a wealth of experimental data of supercritical water (SCW) including structural, dynamical, and electronic properties [3, 4] such that we expect that it should also perform well in the present context of vibrational spectroscopy. Within the realm of AIMD simulations, the computationally much more demanding revPBE0-D3 hybrid functional has been demonstrated to provide an excellent representation of the full-dimensional many-body potential energy surface that describes RTW [16]. Thus, we compare in Fig. S1 in a one-to-one manner also the RDFs of SCW as obtained from RPBE-D3 to the revPBE0-D3 benchmark with favorable agreement at much reduced computational cost due to using a GGA functional instead of a hybrid. When it comes to H-bond dynamics and THz spectroscopy, we refer to direct comparisons of our RPBE-D3 results to NMR relaxation data of SCW (Fig. 3b in the main text) and to the THz spectrum of RTW (Fig. 1a in the main text) with good agreement in both cases.

Using this methodology, we systematically investigate the evolution of the H-bond THz mode upon (isochorically) heating RTW to SCW at RTW density and upon (isothermally) expanding SCW at SCW temperature down to very low densities. To do so, we now accurately compute and fully decompose the THz response at 10 thermodynamic state points at $1.0\,\mathrm{kg\,L^{-1}}$ and temperatures of 300, 350, ..., 700, and 750 K (isochore scan) as well as at 11 state points at 750 K and densities of 0.1, 0.2, ..., 1.0, and $1.1\,\mathrm{kg\,L^{-1}}$ (isotherm scan), thus greatly transcending our earlier work that exclusively focused on structural dynamics [4]; we refer to that publication for a detailed description of the employed simulation protocol.

B. Computational Details

We employed Born-Oppenheimer ab initio molecular dynamics (AIMD) [1] simulations for all reported simulations using the `Quickstep` electronic structure module [17] as implemented in the `CP2k` molecular dynamics simulation package [18]. The RPBE [19, 20] and revPBE0 [19, 21] exchange-correlation functional were computed using the `libxc` package [22] and have been supplemented by adding the D3 dispersion corrections [23] (using the two-body terms and zero damping), thus yielding what we call the RPBE-D3 and revPBE0-D3 functionals. Core electrons were represented using the separable dual-space Gaussian Goedecker-Teter-Hutter type pseudopotentials [24, 25] and the valence electrons were treated using a mixed Gaussian orbital/plane wave (GPW) basis set [26]. We expanded the plane waves up to a cutoff of 500 Ry, whereas the Gaussian orbitals are constructed using the triple-ζ quality TZV2P basis set that includes polarization functions [27] . In case of revPBE0, we employ the auxiliary density matrix (ADM) method [28] and the associated cpFIT3 basis set as provided within the `CP2k` program package [18] to compute the additional non-local Fock exchange contribution in that hybrid functional.

We use a Born-Oppenheimer propagation time step of 0.5 fs and massive Nosé-Hoover-chain thermostatting in case of NVT simulations, whereas all time-dependent properties such as H-bond lifetimes, reorientational relaxation times and in particular all vibrational spectra have been computed from NVE simulations to rigorously avoid any possible artifacts due to thermostatting. The box length of the periodic cubic simulations cell (that hosts 128 water molecules in all simulations) at a given thermodynamic state point is determined

from the selected density. In total, the present study is based on statistics gained by more than 20 ns of AIMD trajectories. More background and details can be found in our previous AIMD studies of SCW [3, 4].

C. Assessing the Density Functional: RPBE-D3 versus revPBE0-D3

In the realm of AIMD studies of liquid water it has been shown that the revPBE0-D3 hybrid functional [19, 21, 23] provides an accurate description of many of its important properties [16, 29–31]. Unfortunately, the computational effort when considering the non-local Fock exchange term in hybrid functionals is increased typically by a factor of about ten compared to GGA functionals, even when using ADM efficiency [28], which is true when comparing revPBE0-D3 to RPBE-D3 for periodic simulations using 128 water molecules. Given that the present study required already more than 20 ns of GGA time (using RPBE-D3) since many thermodynamic state points needed to be investigated on equal footing with exhaustive sampling, using a hybrid functional (such as revPBE0-D3) instead would formally correspond to 200 ns of GGA time and thus induce a formidable computational burden.

Thus, we set out to benchmark our default RPBE-D3 simulations with respect to revPBE0-D3 simulations of SCW in direct comparison to room temperature water (RTW) conditions. The RTW simulation is conducted at 300 K and a density of 0.996 kg L^{-1} such that the box setup exactly coincides with the setup of our RPBE-D3 RTW simulation [11]. We generated a single RTW revPBE0-D3 trajectoy with a duration of 60 ps in the NVT ensemble. In case of SCW where proper statistical sampling is more demanding, we consider 10 independent initial configurations for the revPBE0-D3 benchmark which are identical to those underlying the respective RPBE-D3 NVT simulations. Recall that according to our elaborate SCW sampling protocol [4] we conducted 10 statistically independent NVT simulations for each and every supercritical state point in order to achieve exhaustive sampling since correlation times in SCW can be long. In order to take this issue into account, we thus continued all 10 RPBE-D3 NVT simulations using the revPBE0-D3 functional for 6 ps each, thus resulting in a total simulation time of 60 ps as for RTW.

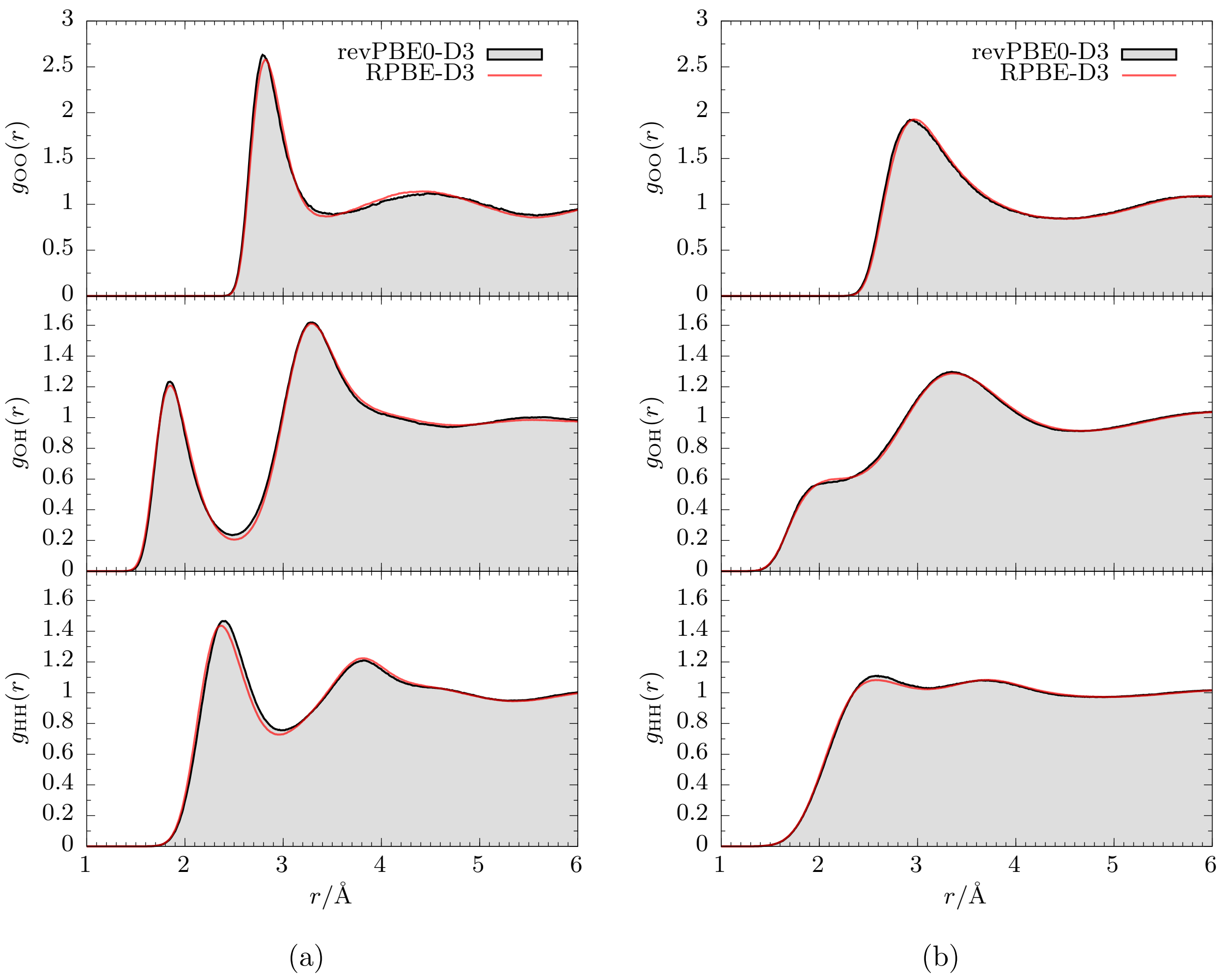

FIG. S1: Radial distribution functions, O–O (top panel), O–H (middle panel) and H–H (bottom panel), of room temperature water (a, RTW) and supercritical water (b, SCW) as given by the RPBE-D3 (red solid line) and revPBE0-D3 (black solid line and shaded area) density functionals. The respective simulations at the two state points have been conducted at the same nominal temperatures and densities, namely at a temperature of 300 K and a density of 0.996 kg L^{-1} in case of RTW and at 750 K and 1.0 kg L^{-1} in case of SCW.

In Fig. S1 we present all RDFs computed from RPBE-D3 (GGA functional) and revPBE0 (hybrid functional) of RTW (panel a) and of SCW at 750 K and 1.0 kg L^{-1} (panel b). Inspecting the figures we observe that the RPBE-D3 and revPBE0-D3 functionals almost perfectly agree regarding the generated structure of RTW and SCW. While such good agreement in case of RTW is already well acknowledged in the literature, see e.g. Ref. 16, it is reassuring that the two functionals also agree on the structure at supercritical conditions.

Since RPBE-D3 agrees with the more sophisticated revPBE0-D3 functional and has been shown previously to reproduce available experimental data on SCW [3, 4], we are now confident that it can also correctly describe the vibrational spectroscopy of SCW.

D. Van der Waals Reference Simulations: Corresponding States of RPBE-D3 Water and the Lennard-Jones Fluid

In the main text we compare our RPBE-D3 simulations of SCW with a simple Lennard-Jones fluid model, dubbed LJ-wat therein since the LJ parameters are those describing the oxygen-oxygen interactions according to the SPC water model, in order to disentangle effects exclusively due to isotropic van der Waals interactions from those imposed in addition by the directional H-bonding interactions. To meaningfully allow for such comparison, the corresponding thermodynamic state points of the two very different computational descriptions of water need to be comparable. In Fig. S2 we display the phase diagrams of water given by the experimental IAPWS95 equation of state [32] and of the standard LJ fluid [33] in panels (a) and (b), respectively. Note that the critical point (CP) of RPBE-D3 water [3] has been located at about $T_\mathrm{c} = 710\,\mathrm{K}$ and $\rho_\mathrm{c} = 0.31\,\mathrm{kg\,L^{-1}}$. The finite-size corrected CP of the LJ fluid [34] is located at $T_\mathrm{c} = 1.312\ \varepsilon/k_\mathrm{B}$ and $\rho_\mathrm{c} = 0.316/\sigma^3$; as usual this CP is reported using the natural energy and length units of the LJ potential, which are uniquely defined in terms of ε and σ, respectively, thus being universal for all LJ fluids (of course including our "LJ-wat water").

Next, according to the principle of corresponding states [35], two thermodynamics states are thermodynamically comparable if their reduced thermodynamic field variables are equal with respect to their corresponding CPs. In our case this means that the reduced temperature $T^* = T/T_\mathrm{c}$ and the reduced density $\rho^* = \rho/\rho_\mathrm{c}$ must be equal. Using $T_\mathrm{c} = 1.312\ \varepsilon/k_\mathrm{B}$ for the LJ fluid, we obtain a temperature of $1.44\ \varepsilon/k_\mathrm{B}$ for the isotherm corresponding to $T^* = T/T_\mathrm{c} = 1.056$. At this temperature we selected 10 state points at different densities, namely at 0.10, 0.18, 0.27, 0.35, 0.43, 0.52, 0.60, 0.68, 0.77, and $0.85/\sigma^3$ to perform the supercritical isotherm scan of the LJ fluid as depicted by the up-triangles in Fig. S2(b). To find a comparable isochore we opted to select the triple point density of the LJ fluid [33] of $0.85/\sigma^3$ because the corresponding RPBE-D3 simulations are also carried out close to the triple point density of water. We then selected 8 state points on that isochore to increase the

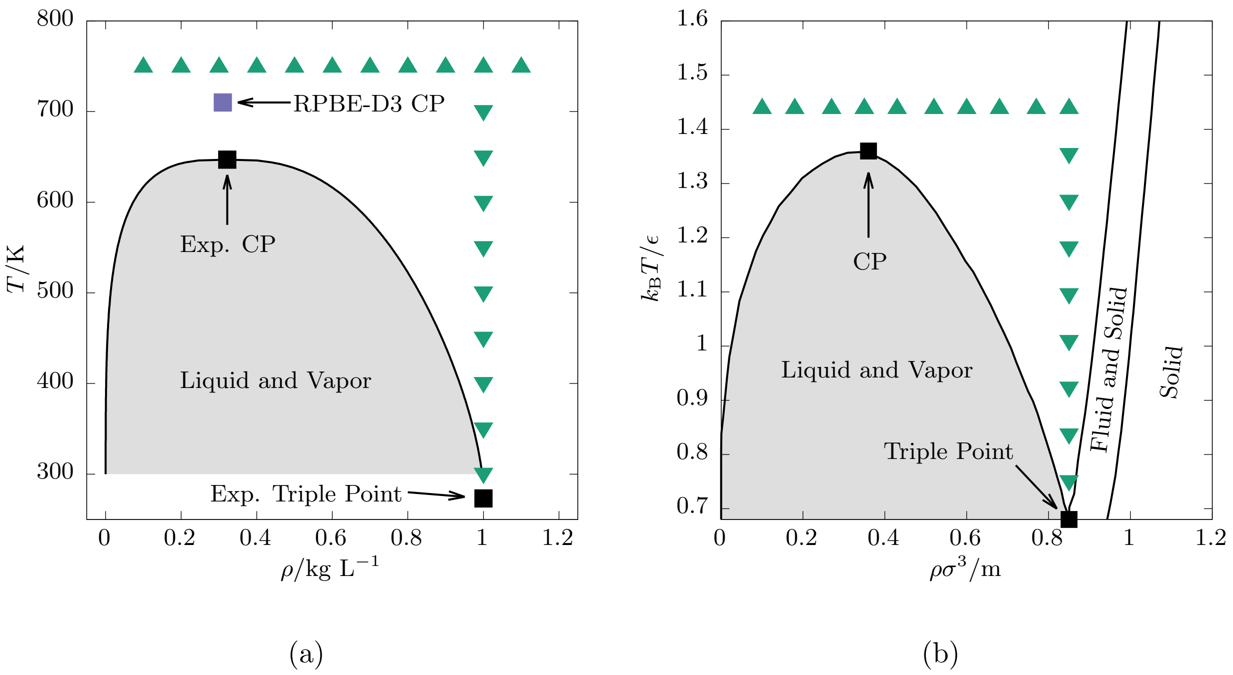

FIG. S2: Phase diagrams of water as given by the accurate experimental IAPWS95 equation of state [32] in panel (a) and of the Lennard-Jones fluid reproduced from Ref. 33 in panel (b) as black solid lines and gray areas on which the respective critical and triple points are marked by black solid squares. Note that the entire coexistence curve of the LJ fluid [33] is not finite-size corrected and thus the location of the critical point differs from the one obtained after its finite-size scaling [34] which is used for scaling (see text). The critical point of RPBE-D3 water in panel (a) has been obtained by ab initio Gibbs Ensemble Monte Carlo simulations [3] and is located at $T_c = 710\,\mathrm{K}$ and $\rho_c = 0.31\,\mathrm{kg\,L^{-1}}$ (purple filled square). The density scan along the supercritical isotherm is conducted at $T^* = T/T_c = 1.056$, corresponding to temperatures of $750\,\mathrm{K}$ and $1.44\ \varepsilon/k_B$ in case of RPBE-D3 water and the LJ fluid, respectively, and are marked using green up-triangles. The temperature scan along the isochore is conducted at the triple point density, corresponding to $0.9998\,\mathrm{kg\,L^{-1}}$ and $0.85/\sigma^3$ in case of water and the LJ fluid taken from Refs. 32 and 33, respectively, and are marked using green down-triangles.

temperature up to its value at the isotherm, namely 0.75, 0.836 25, 0.9225, 1.008 75, 1.095, 1.181 25, 1.2675, 1.353 75, and 1.44 ϵ/k_B as marked using the down-triangles in Fig. S2(b). Note that the last state point lays on both, the supercritical isotherm and the isochore scan akin to what we did for RPBE-D3 water as visualized in Fig. S2(a).

All conducted simulations of the LJ fluid and RPBE-D3 water are summarized in Fig. S2, thus demonstrating that we indeed constructed a comparable isochore and isotherm that probe corresponding regions in the full phase diagram. The isochore probes thermal activation effects from about the triple point (where the two fluids are in their liquid state) up to the respective supercritical phase at the same reduced temperature. Once arrived in the supercritical phase, the isotherm spans the regime from the rather high, "liquid-like" isochore density towards very low, "gas-like" densities [2].

II. SPECTRAL ANALYSES

A. Computing Linear Absorption Coefficients

It is well known that the Beer-Lambert absorption coefficient (or linear absorption cross section)

$$\alpha(\omega) = \frac{\beta\pi}{3cV\epsilon_0 n(\omega)}\mathcal{F}\left[\left\langle \dot{\vec{M}}(0)\dot{\vec{M}}(t)\right\rangle\right] \tag{S1}$$

is computed as the Fourier transform of the time auto-correlation function of the total dipole moment velocity $\dot{\vec{M}}(t)$, where ω is the angular frequency (whereas we report all spectra in wavenumbers $\widetilde{\nu}$ in the main text as commonly done), $n(\omega)$ the frequency-dependent refractive index, c the speed of light in vacuum, ϵ_0 the vacuum permittivity, $\beta = 1/k_\mathrm{B}T$, k_B the Boltzmann constant, T the temperature, V the volume, and $\mathcal{F}[\cdots]$ denotes a forward Fourier transform. This formula provides the proper classical limit of the underlying exact quantum linear response expression and embodies what is often called the "harmonic quantum correction factor" [36]. The equation is given in SI units and $\alpha(\omega)$ is the natural Beer-Lambert absorption coefficient, i.e. defined via the natural logarithm [37]. The molar absorption coefficients that we report in the main text for one-to-one comparison to experiments,

$$\kappa(\omega) = \alpha(\omega)/\frac{\rho}{M} \ , \tag{S2}$$

TABLE I: Refractive indices, n_{gap}, at a wavelength of 589 nm ($\approx 17\,000\,\text{cm}^{-1}$), see text, for all simulated supercritical water state points at 750 K and various densities, ρ, given by the IAPWS95 equation of state [32, 40, 41].

ρ / kg L^{-1}	n_{gap}	ρ / kg L^{-1}	n_{gap}	ρ / kg L^{-1}	n_{gap}
0.1	1.031	0.5	1.162	0.8	1.261
0.2	1.064	0.6	1.195	0.9	1.293
0.3	1.096	0.7	1.228	1.0	1.325
0.4	1.129			1.1	1.356

are obtained from the absorption coefficients by dividing by the molar concentration, where ρ is the volumetric mass density at the respective thermodynamic state point and M is the molar mass of a water molecule.

We compute the frequency-dependent refractive index $n(\omega)$, which is required according to Eq. (S1) to access $\alpha(\omega)$ as experimentally measured, by applying the Kramers-Kronig relations in full analogy to what has been done before by others as well as by ourselves [38, 39]. To do so, the refractive index in the optical gap between the highest vibrational absorption (ignoring overtones) and the first electronic absorption, n_{gap}, needs to be known. This refractive index strongly depends on the thermodynamic state point, i.e. temperature, pressure, and in particular the density. The respective values for n_{gap} are therefore obtained from the IAPWS95 equation of state at 589 nm ($\approx 17\,000\,\text{cm}^{-1}$) [32, 40, 41] for each simulated thermodynamic state point and summarized in Table I. We also use the IAPWS95 equation of state to obtain the respective refractive indices along our isochore scan at 1.0 kg L^{-1}. However, temperature changes at constant density affect the refractive index only little, being 1.333 at 300 K and 1.325 at 750 K.

As commonly done when computing spectra from the Fourier transform of time-correlation functions, we employ a window function (here we use the well-known Hann window) to smoothen our spectra which is elaborately explained in Sec. 13.4 of Ref. 42. We consistently employ the same smoothing level for all shown spectra throughout the main text and the SI. In Fig. S3 we exemplarily compare the smoothened spectra as presented in the main text for RTW and SCW at 750 K and 0.1 kg L^{-1} to the corresponding raw data as directly provided by the Fourier transform of the finite trajectories. The raw spectra can be used

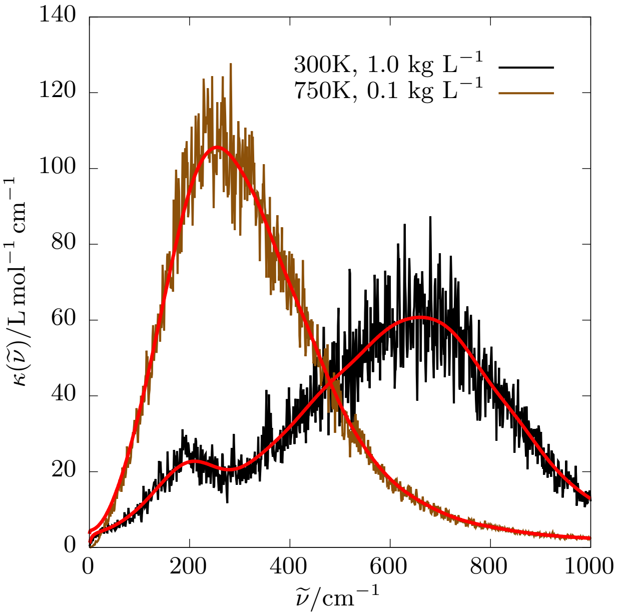

FIG. S3: Raw molar THz absorption coefficients $\kappa(\widetilde{\nu})$ of room temperature water (black line) and of supercritical water at 750 K and 0.1 kg L^{-1} (brown line). The corresponding smoothened spectra (see text for description) as reported in the main text are shown as red lines in both cases.

to assess the statistical uncertainty of the reported lineshapes which is still present despite using as many as 1 600 000 simulation steps at each state point (corresponding to 0.8 ns of AIMD time). Importantly, the Hann smoothened spectra perfectly reproduce the features of the corresponding raw spectrum without any further adjustments given the trajectory length.

B. Hindered Translations: Two-Body Vibrational Density of States

In the main text, we employed a projected relative velocity [43], $\Delta v_{IJ}(t)$ defined by Eq. (1) therein, in order to capture the relative motion of two water molecules I and J and computed the corresponding two-body vibrational density of states (2B-VDOS) $\Lambda^{2B}(\widetilde{\nu})$, as defined in Eq. (2) therein; recall that any vibrational DOS probes the particle dynamics and not dipolar fluctuations implying that its intensity can be scaled to a useful reference.

Since $\Delta v_{IJ}(t)$ is computed for water pairs $I - J$, the resulting 2B-VDOS clearly depends on which water pairs are selected from the trajectory. In Fig. S4a, we compute it using

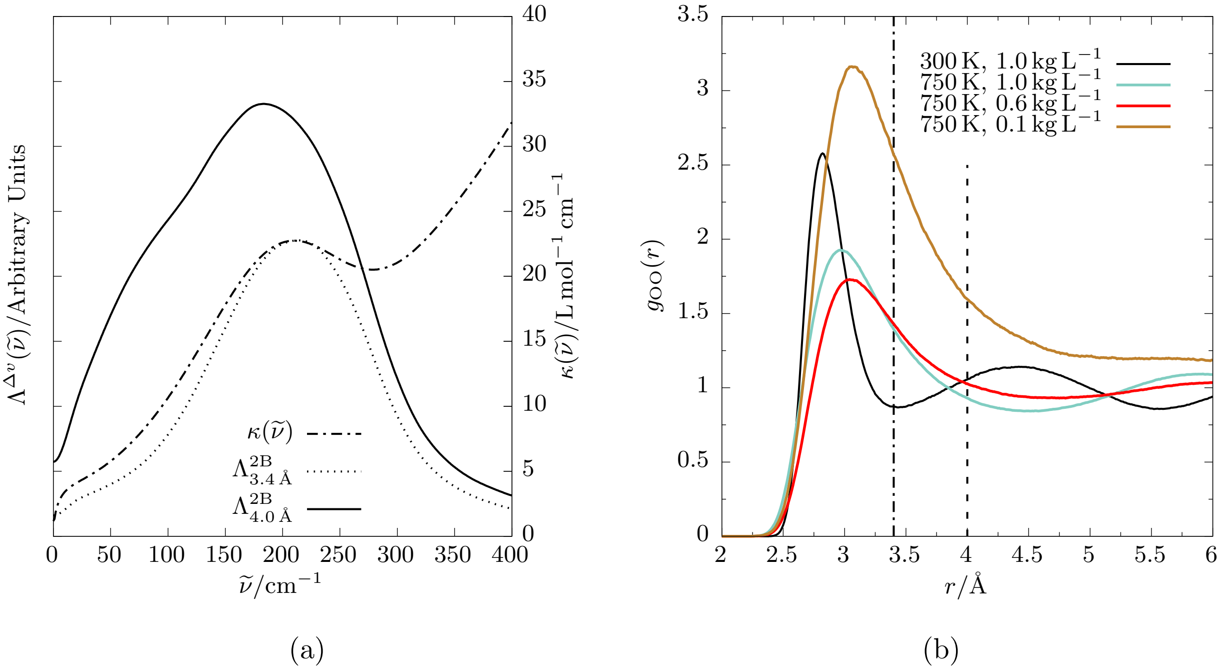

FIG. S4: (a) Two-body spectral density ($\Lambda^{2B}(\widetilde{\nu})$, Eq. (2)) from RPBE-D3 simulations of RTW obtained when applying $O_I \cdots O_J$ water pair cutoff distances of 3.4 ($\Lambda^{2B}_{3.4\,Å}(\widetilde{\nu})$: dotted line) and 4.0 Å ($\Lambda^{2B}_{4.0\,Å}(\widetilde{\nu})$: solid line) as used throughout in the main text. The full THz spectrum of RTW ($\kappa(\widetilde{\nu})$, right scale) is included as a black dashed-dotted line for reference and $\Lambda^{2B}_{3.4\,Å}(\widetilde{\nu})$ is scaled such that its maximal intensity corresponds to the maximal intensity of $\kappa(\widetilde{\nu})$ whereas $\Lambda^{2B}_{4.0\,Å}(\widetilde{\nu})$ is scaled by the very same factor to retain the relative intensities of these two 2B-VDOS spectra. (b) Oxygen-oxygen radial distribution functions of RTW (black line) and of SCW at 750 K and densities of 0.1 (brown line), 0.6 (red line), and 1 kg L^{-1} (green line). The vertical dashed–dotted and dashed lines mark the OO cutoff criteria of 3.4 and 4.0 Å between water pairs as employed to compute $\Lambda^{2B}_{3.4\,Å}(\widetilde{\nu})$ and $\Lambda^{2B}_{4.0\,Å}(\widetilde{\nu})$, respectively.

$O_I \cdots O_J$ cutoff distances of 3.4, $\Lambda^{2B}_{3.4\,Å}(\widetilde{\nu})$, and 4.0 Å, $\Lambda^{2B}_{4.0\,Å}(\widetilde{\nu})$, respectively; recall that no angular criterion depending on the relative orientation of the two water molecules is applied for reasons explained in the main text. For direct reference, we also show in panel (a) the molar absorption coefficient of water at ambient conditions, $\kappa(\widetilde{\nu})$, reported in absolute units that are defined at the right scale. Since absolute intensities of $\Lambda^{2B}(\widetilde{\nu})$ are not defined we scaled $\Lambda^{2B}_{3.4\,Å}(\widetilde{\nu})$ such that its maximal intensity corresponds to the maximal intensity of $\kappa(\widetilde{\nu})$ of RTW whereas $\Lambda^{2B}_{4.0\,Å}(\widetilde{\nu})$ has been scaled by the very same factor to retain the relative intensities of these two 2B-VDOS spectra.

We observe that upon applying the strict water-water pair distance criterion of 3.4 Å, thus yielding $\Lambda^{2B}_{3.4\,\text{Å}}(\widetilde{\nu})$, almost exactly recovers at the level of the two-body vibrational density of states spectrum the intermolecular H-bond peak as seen in the total THz spectrum $\kappa(\widetilde{\nu})$. In contrast, the peak of $\Lambda^{2B}_{4.0\,\text{Å}}(\widetilde{\nu})$ is a bit red-shifted and features a small shoulder around $75\,\text{cm}^{-1}$. The reason is that we opted to use a OO cutoff distance of 4.0 Å consistently for all sub- and supercritical state points. In case of all SCW states considered, that larger water-water distance ensures that the first peak of the OO radial distribution function is largely included (see Fig. S4b) when selecting all adjacent water pairs for the 2B-VDOS analysis. However, in the case of RTW, that OO cutoff obviously exceeds the first RDF minimum (see Fig. S4b) meaning that quite some contributions of the second peak, and thus of water pairs where one molecule belongs already to the second solvation shell, are included when computing $\Lambda^{2B}(\widetilde{\nu})$.

Moreover, RTW also features so-called interstitial water molecules [11] which are in close vicinity of the reference molecule but do not form a H-bond with it, thus contributing pair distances to the O–O RDF beyond its first minimum and thus beyond roughly 3.4 Å. Using the larger cutoff of 4.0 Å therefore adds all these non H-bonded contributions to the corresponding spectral density $\Lambda^{2B}_{4.0\,\text{Å}}(\widetilde{\nu})$. The corresponding hindered translations and thus the associated intermolecular vibrations generally occur at somewhat lower frequencies than those due to H-bonded water pairs, and thus effectively red-shift $\Lambda^{2B}_{4.0\,\text{Å}}(\widetilde{\nu})$ with respect to $\Lambda^{2B}_{3.4\,\text{Å}}(\widetilde{\nu})$. These particle motions are, of course, also present when computing the total THz spectrum $\kappa(\widetilde{\nu})$. However, we know that they are "THz-silent", i.e. their intermolecular oscillator strength and thus contribution to the total THz response is negligible as analyzed in detail in Refs. 15, 44, whereas they obviously contribute to the intensity of the vibrational density of states and thus lead to the aforementioned shoulder in RTW when using the larger cutoff as required to consistently analyze SCW. Overall, the effect of both, the red-shift and the shoulder of the 2B-VDOS peak compared to the THz spectrum is entirely negligible in the context of our $\Lambda^{2B}(\widetilde{\nu})$ analysis in the main text.

C. Hindered Rotations: Librational Density of States

To identify the H-bond mode in the THz spectrum we employ the two-body VDOS, $\Lambda^{2\mathrm{B}}(\widetilde{\nu})$, as described in the main text. In addition, we also use a dissection of atomic velocities to separate off the librational band. Since this band emerges due to hindered rotations, we can compute the corresponding spectral density from the rotational velocities of each atom in the context of the respective water molecule. The rotational velocity of each atom can be obtained by calculating the angular momentum of each molecule

$$\vec{L}_I(t) = \sum_{I_i} m_{I_i} \left[\vec{r}_{I_i}(t) \times \left(\vec{v}_{I_i}(t) - \vec{v}_I^{\,\mathrm{trans}}(t) \right) \right] \quad , \tag{S3}$$

where $\vec{r}_{I_i}(t)$ is the position of the ith atom in molecule I with respect to its center of mass and $\vec{v}_{I_i}(t)$ is the corresponding velocity at time t, while $\vec{v}_I^{\,\mathrm{trans}}(t)$ denotes the center of mass velocity of molecule I due to its purely translational motion. This subtraction implies that the center of mass velocity is explicitly excluded while computing the angular momentum. From that angular momentum, the corresponding angular velocity

$$\vec{\omega}_I(t) = \mathbf{I}_I^{-1}(t) \vec{L}_I(t) \tag{S4}$$

of molecule I can be calculated, where $\mathbf{I}_I^{-1}(t)$ is the inverse of its moment of inertia

$$\mathbf{I}_I(t) = \sum_{I_i} m_{I_i} \left[\vec{r}^{\,2}_{I_i}(t)\mathbf{E} - \vec{r}_{I_i}(t) \otimes \vec{r}_{I_i}(t) \right] \quad , \tag{S5}$$

where $\mathbf{E}$ is the identity matrix and $\otimes$ denotes the tensor product. Again, the atomic positions $\vec{r}_{I_i}(t)$ are expressed with respect to the corresponding center of mass of molecule I. For the rotational velocity of each atom i within molecule I then follows

$$\vec{v}_i^{\,\mathrm{rot}}(t) = \vec{\omega}_I(t) \times \vec{r}_{I_i}(t) \quad . \tag{S6}$$

The corresponding spectral density

$$\Lambda^{\mathrm{rot}}(\omega) = \mathcal{F}\left[\sum_i^{N_{\mathrm{atoms}}} \left\langle \vec{v}_i^{\,\mathrm{rot}}(0) \vec{v}_i^{\,\mathrm{rot}}(t) \right\rangle \right] \quad , \tag{S7}$$

is obtained from the autocorrelation function of $\vec{v}_i^{\,\mathrm{rot}}(t)$ after averaging over all atoms, where $\mathcal{F}\left[\cdots\right]$ denotes a forward Fourier transform. Note that the intensity of that spectral density is arbitrary and usually scaled to some convenient reference.

Using $\Lambda^{\rm rot}(\widetilde{\nu})$ and $\Lambda^{\rm 2B}(\widetilde{\nu})$ together, the total THz spectrum can be faithfully assigned to hindered rotational motion (librations) and hindered translational motion (*intermolecular* vibrations) of water pairs, respectively. However, since these two spectral densities are based on particle dynamics only, the intensities cannot directly be compared to the true THz spectrum (which is weighted by the frequency-dependent dipolar oscillator strength) and, therefore, can be scaled to some convenient reference. This implies that we can indeed determine where the two different kinds of molecular motions occur in the THz spectrum, but we cannot tell how much dipolar intensity is ascribed to each of them.

An alternative method that allows one to separate the (single-water) librational band from the (water-pair) H-bond stretching contributions, while retaining the correct dipolar (IR/THz) intensities is our so-called Cross Correlation Analysis (CCA) technique [45]. In a nutshell, the CCA dissects the THz (or full IR) spectrum in terms of distinct self- and cross-terms, e.g. based on topological connectivity criteria. Using the CCA approach, it was possible to dissect the spectrum of aqueous solutions of both, ions and molecules and to assign the spectral features to distinct solvation shells [45–47].

In the present context of SCW, we apply a tailored version of the CCA technique and use a single group, containing all water molecules, and employ no further relation between the molecules. The formalism then leads to the following dissection in the present case

$$\kappa(\omega) = \kappa^{\rm A}(\omega) + \kappa^{\rm C}(\omega) \ , \tag{S8}$$

where $\kappa^{\rm A}(\omega)$ contains the self-term of the individual water molecules, and $\kappa^{\rm C}(\omega)$ encompasses the cross-terms between all water pairs. In this context, we could establish recently [45, 47] that the librational band is overwhelmingly contained in $\kappa^{\rm A}(\omega)$. This technique thus allows us to effectively separate the librational band from the THz spectrum including the correct dipolar (IR/THz) intensities in contrast to any vibrational density of states that exclusively probes particle dynamics.

Using this approach, we present in Fig. S5 the THz spectra $\kappa(\widetilde{\nu})$ together with the self-term $\kappa^{\rm A}(\widetilde{\nu})$ as a result of the CCA decomposition, as well as the spectral densities due to the rotational dynamics of the H atoms $\Lambda^{\rm rot}(\widetilde{\nu})$ as defined in Eq. (S7) and due to two-body translational dynamics $\Lambda^{\rm 2B}(\widetilde{\nu})$ as defined in Eq. (2) in the main text. In case of RTW (upper left panel), the total THz spectrum shows two distinct peaks centered around roughly 200 and 650 $\rm cm^{-1}$. These two signals are nicely reproduced by $\Lambda^{\rm 2B}(\widetilde{\nu})$ and $\Lambda^{\rm rot}(\widetilde{\nu})$, respectively,

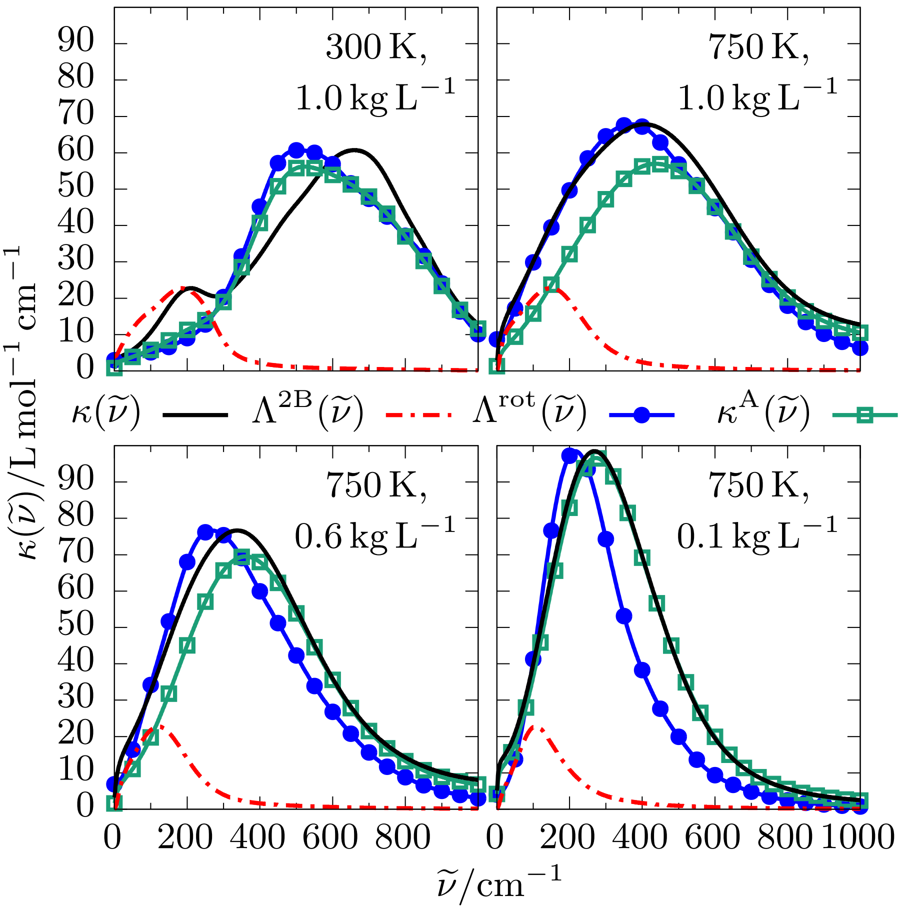

FIG. S5: Molar absorption coefficients $\kappa(\tilde{\nu})$ (solid black lines) of room temperature water (RTW, upper left panel) and supercritical water at 750 K and densities of 0.1 (lower right panel), 0.6 (lower left panel), and 1.0 kg L^{-1} (upper right panel) from RPBE-D3 simulations. The self-term $\kappa^{A}(\tilde{\nu})$ (solid green line with squares) as well as the spectral densities due to the hindered rotational dynamics (see text) $\Lambda^{rot}(\tilde{\nu})$ (blue solid with circles) and due to two-body hindered translational motion (see main text) $\Lambda^{2B}(\tilde{\nu})$ (red dashed–dotted lines) are also shown. The intensities of $\Lambda^{rot}(\tilde{\nu})$ and $\Lambda^{2B}(\tilde{\nu})$ are scaled (see text) such that their maxima correspond to the maximum intensity of the respective librational band and H-bond stretch mode in $\kappa(\tilde{\nu})$. In case of SCW, the H-bond peak (nicely visible in RTW) is not visible in the THz spectrum, therefore the same scaling of $\Lambda^{2B}(\tilde{\nu})$ used for room temperature water is consistently applied for all supercritical states for illustrative reasons. Clearly this overestimates the contribution of $\Lambda^{2B}(\tilde{\nu})$ in SCW, since the overall number of water pairs decreases with density, which implies that the contributions to the THz spectrum due to two-body hindered translations (*intermolecular* vibrations) should decrease accordingly.

and therefore can indeed be assigned to the intermolecular H-bond stretch and the librational modes of the water molecules, respectively. Moreover, $\kappa^{\mathrm{A}}(\widetilde{\nu})$ also closely reproduces the librational band and almost exactly maps the entire shape of $\Lambda^{\mathrm{rot}}(\widetilde{\nu})$. However, recall that the lineshape of $\kappa^{\mathrm{A}}(\widetilde{\nu})$ contains true dipolar (IR/THz) intensities and is not scaled at all, whereas $\Lambda^{\mathrm{rot}}(\widetilde{\nu})$ is scaled to a convenient value, in this case to the maximum intensity of $\kappa(\widetilde{\nu})$.

Turning now our attention to SCW, we find that the librational band given by $\kappa^{\mathrm{A}}(\widetilde{\nu})$ and $\Lambda_{\mathrm{rot}}(\widetilde{\nu})$ significantly red-shifts as a function of temperature and even red-shifts furthermore when the density is decreased at the supercritical temperature of 750 K, see Fig. S5. According to $\Lambda^{\mathrm{2B}}(\widetilde{\nu})$, the hindered two-body vibrations of water pairs follow the same trend and systematically red-shift as a function of increasing temperature and decreasing density from $\approx 180\,\mathrm{cm}^{-1}$ at RTW to $\approx 100\,\mathrm{cm}^{-1}$ at 750 K and $0.1\,\mathrm{kg\,L}^{-1}$ in SCW. Regarding the dipolar (IR/THz) intensities, we rely on $\kappa^{\mathrm{A}}(\widetilde{\nu})$ which contains that correct oscillator strengths stemming from librational motion. From $\kappa^{\mathrm{A}}(\widetilde{\nu})$, we conclude that the THz spectrum of SCW almost entirely consists of the librational band and it follows that the intensity due to the *intermolecular* vibrations of water pairs only contributes little to the total IR absorption.

D. Mid-IR Spectroscopy: Intramolecular O-H Stretch

A well-established method to assess H-bonding in water, which is used since decades up to the present day to also analyze supercritical fluids [48–50], is mid-IR spectroscopy of the intramolecular O−H stretching band as discussed in more detail in Sec. IV. Within an H-bonded environment, the intramolecular O−H bond is very systematically weakened due to the presence of intermolecular H···O bonds to other molecules. Therefore, it is well established that the peak location of the O−H stretching band in the mid-IR frequency regime systematically red-shifts to lower values as a function of the intermolecular H-bond strength [51–54]. Besides, the presence of H-bonds in the liquid not only affects the position of the O−H stretching band, but also its intensity. In this context, is has been demonstrated that the transition dipole moment dramatically increases in a H-bonded liquid with the consequence that the intramolecular O−H stretching band in liquid water appears “brighter” than in the gas phase [50, 55, 56].

Since the AIMD simulations we performed provide access to the full IR frequency range, thus including not only far-IR / THz but also mid-IR frequencies, it is worthwhile to analyze the intramolecular O−H stretching peak on equal footing with the THz peak, which clearly remains the focus of the present investigation. However, when it comes to using AIMD to compute IR absorption coefficients, it must be kept in mind that the technique relies on using classical nuclear dynamics to generate trajectories in phase space along which the dipole moment is computed on-the-fly from electronic structure [1]. The Fourier transform of the classical time auto-correlation function of the dipole moment function finally provides the IR absorption coefficient as sketched in Sec. II A. However, the quantum-mechanically rigorous procedure requires the quantum time auto-correlation function of the dipole moment operator instead. There is a vast literature on how to approximately take nuclear quantum effects into account when computing IR spectra from classical trajectories as summarized for instance in Ref. 36 and comprehensively reviewed in a broader context in Ref. 57. Upon analyzing commonly used so-called "quantum correction factors" makes clear that they systematically affect the computed IR intensities as a function of frequency [36]. Unfortunately, these intensity modifications strongly depend on the variant used, in particular as frequencies increase toward the mid-IR regime, meaning that absolute intensities are not rigorously accessible when computing IR absorption coefficients based on classical trajectories. This is particularly true for the O−H stretching band where the underlying oscillators are stiff, implying significant zero-point motion and thus a very pronounced quantum-mechanical character for these intramolecular resonances at mid-IR frequencies. This is the reason why sophisticated mixed quantum-classical vibrational spectroscopy methods have been developed long ago to successfully compute the lineshape of the O−H stretching peak in water [55], as recently even demonstrated for D_2O-doped supercritical water [50]; we refer the interested reader to these references for access points to the vast literature in that field. In the context of the present study with its clear focus on far-IR / THz frequencies, we note that the opposite, namely the classical limit, is eventually reached for soft intermolecular modes, which are therefore faithfully reproduced by AIMD as proven for liquid water by one-to-one comparison of its THz spectrum to experiment in Fig. 1a of the main text. We therefore restrict the discussion in this section to the relative frequency shift of the position of the O−H stretching band as given by its maximum on the frequency axis.

In Fig. S6 we present the red-shift $\Delta\widetilde{\nu}$ of the intramolecular O−H stretching band in SCW

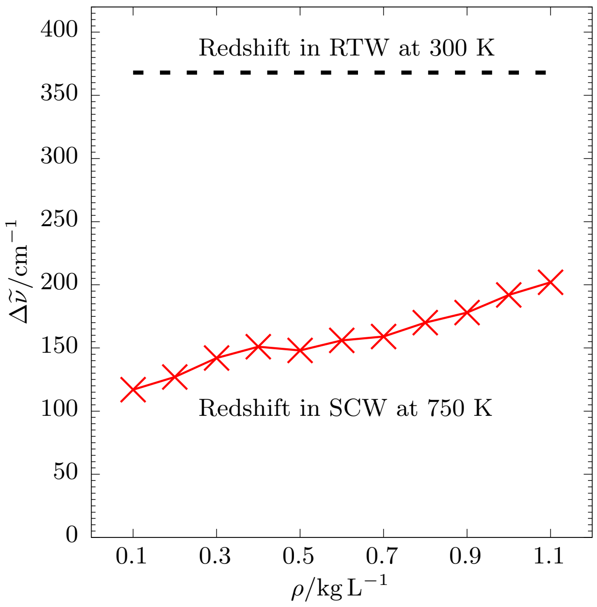

FIG. S6: Red-shift of the intramolecular O−H stretching band, $\Delta\tilde{\nu}$, based on the frequency of maximal absorption in SCW at 750 K as a function of density with respect to a single gas-phase water molecule at the same temperature (red crosses); the red solid line connects the data linearly and serves only as a guide to the eye. For one-to-one reference, the same red-shift in case of liquid water at ambient conditions, RTW, also with respect to a single gas-phase water molecule at the same temperature, i.e. $T = 300\,\mathrm{K}$, is shown as a black dashed horizontal line. The frequencies of maximal absorption of the water monomers at 300 and 750 K are located at 3790 and 3741 cm^{-1}, respectively.

with respect to an isolated gas-phase water molecule at the same temperature of 750 K to provide an internally consistent gauge to quantify the frequency shift using the same computational protocol and parameters as before. For one-to-one reference, we also show the red-shift in case of liquid water at ambient conditions, RTW, with respect to a single water molecule, now computed at 300 K to stay consistent. In both cases, the calculated shift is based on the frequency of maximal absorption of the O−H stretching band at the respective thermodynamic state point.

Inspecting now the data in Fig. S6, we observe the expected very pronounced red-shift of the O−H stretching peak in case of RTW, which is well known in the literature [51–54] to indicate the presence of the H-bond network that characterizes liquid water at ambient conditions. Turning next to SCW, the O−H red-shift is found to be dramatically reduced

even at the highest densities which are comparable to RTW. This is surprising because at a density as high as $1.1\,\mathrm{kg\,L^{-1}}$ the average coordination number (determined by integrating the O–O RDF) is above 6 on average [4]. In such a highly coordinated state, one would expect to observe an even more pronounced red-shift than in RTW – provided that this fluid is H-bonded and not simply dense! In other words: The O−H stretching band should red-shift much more dramatically than observed in order to support the presence of H-bonding in particular at such high-density states. We note that the same conclusion concerning a much reduced O−H red-shift in SCW as obtained here from AIMD was drawn earlier in a recent, sophisticated quantum-classical study focusing exclusively on the intramolecular stretching vibrations of SCW as seen in the mid-IR frequency window [50].

Moreover, the average coordination number at the lowest density considered, $0.1\,\mathrm{kg\,L^{-1}}$, is below 1 on average [4]. In terms of the coordination number, this low-density SCW state and the high-density SCW state at $1.1\,\mathrm{kg\,L^{-1}}$ can therefore be regarded as "gas-like" and "liquid-like" states, respectively, in a loose sense. If there was substantial H-bonding in "liquid-like" SCW one would – conversely – expect a much more prominent red-shift $\Delta\widetilde{\nu}$ as a function of increasing density when going from low- to high-density SCW compared to the one we observe in Fig. S6 (in particular if that change is judged on the scale set by the red-shift in RTW at a similarly high density). Thus, our additional systematic investigation of the intramolecular O−H stretching band in SCW as a function of density compared to that observed in RTW is fully consistent with the conclusion as presented in the main text and concisely announced by the title of this publication.

We could close the discussion of mid-IR spectroscopy at this point given the dramatically suppressed red-shift of the O−H stretching band observed in SCW compared to RTW. However, one might even refine and strengthen our arguments by asking if that small red-shift in SCW could be understood in terms of the underlying structural dynamics, and why it varies as a function of the density the way it does. Thus, we are going to connect in what follows the O−H red-shift (see Fig. S6) with the intermolecular orientational arrangement of water dimers in SCW compared to RTW, including also its density dependence within SCW (see Fig. S7) as analyzed in more detail in the next section. Recall that the shift of the intramolecular O−H stretching band is known since long to correlate very well with the intermolecular O$\cdots$O and O$\cdots$H distances as well as with the intermolecular O−H$\cdots$O angle [51–54]. In particular, a shorter distance and an ideal linear H-bond arrangement per-

fectly correlate with a stronger red-shift of the O−H stretch. Water molecules in SCW still have close neighbors within the typical H-bond distances found in RTW, since the average coordination number is only somewhat below 1 in case of $0.1\,\mathrm{kg\,L^{-1}}$ and even exceeds 6 in case of $1.1\,\mathrm{kg\,L^{-1}}$. Therefore, there certainly exist instantaneous water-water pair arrangements in SCW which are characterized by reasonably short O···H distances and close to linear O−H···O angles which thus correspond to transient H-bond contacts. Since the intramolecular O−H stretching vibration is very fast given its oscillation period, mid-IR spectroscopy must detect such short-lived contacts even if they are only fleetingly existing (see main text and Sec. IV for more extensive discussion of that aspect). But what is the population and thus the relative importance of such water-water contacts with reasonably short O···H distances and close to linear O−H···O angles in SCW in a statistical sense?

The probability of finding certain intermolecular O···H distances together with specific O−H···O angles is captured by the joint angle-distance distribution functions depicted later in Fig. S7. Comparing RTW (see Fig. S7a) with SCW at $1.0\,\mathrm{kg\,L^{-1}}$ (see Fig. S7b) we find that the intermolecular O···H distance increases only slightly. More importantly however, the distribution of the O−H···O angle dramatically blurs towards wider, non-linear angles in the supercritical state, which is perfectly reflected by the O–H RDF (inset of Fig. S7b) where the prominent H-bond feature around 1.85 Å known from RTW completely vanishes in SCW in favor of an insignificant shoulder; we refer to Ref. 4 for an elaborate discussion of the O–H RDF. In other words: The joint distribution functions (see Fig. S7), which quantify the intermolecular water-water structure in terms of the two relevant H-bonding coordinates, namely intermolecular distance and angle, show that SCW does no longer feature the pronounced preference of close to linear O−H···O angles known from RTW, but very non-linear arrangements instead – even if the O···H distances are sufficient short! Such enormously flattened angle distributions can be traced back to the enhanced thermal fluctuations in SCW resulting in dramatically reduced H-bond lifetimes and reorientational relaxation times, namely from roughly 2500 fs in RTW to ≈ 60 fs in SCW at $1.0\,\mathrm{kg\,L^{-1}}$ according to Fig. 3a. Indeed, the probability to find quasi-linear O−H···O angles for water dimers at minimal intermolecular distance is as high as ≈ 80 % in case of RTW, thus ambient water clearly is a H-bonded liquid. In the supercritical state, the overwhelming majority of the O−H···O angles turns out to strongly deviate from 180° (with the probability to encounter quasi-linear O−H···O angles being as low as ≈ 40 to 30 % for SCW at 1.1 and $0.1\,\mathrm{kg\,L^{-1}}$,

respectively, using the same approach). This implies that SCW, even at densities exceeding that of RTW, is dominated by strongly bent, non-linear water-water orientations w.r.t. the $O\cdots O$ axis which do not qualify to be called H-bonds according to widespread use of the term in the context of bulk water [58]. The direct connection from these structural findings back to mid-IR spectroscopy and thus to the O−H stretch is established since long in the literature [51–54]. Therein, both structural changes as we observe them in SCW, i.e. elongated $O\cdots H$ distances and strongly non-linear $O{-}H\cdots O$ angles, have been shown [51–54] to be the structural reason underlying a significantly decreased red-shift of the O−H stretching band, which is exactly what we observe in our mid-IR spectra when going from RTW to SCW.

Finally, turning to the evolution of the O−H shift observed along the supercritical isotherm we find that the red-shift increases as a function of increasing density. This can again be understood in terms of the changing intermolecular angle and distance joint distribution functions (see Fig. S7b-d). Along the isotherm, we observe that the intermolecular $O\cdots H$ distance systematically increases and that the $O{-}H\cdots O$ angle distribution even further flattens as a function of decreasing density. Both structural changes along the isotherm can simply be understood in terms of the macroscopic density which naturally affects the packing of the water molecules. At high densities the water molecules are forced to move closer together, obviously resulting in shorter intermolecular distances. Moreover, the rotation of the molecules is also more hindered at higher densities because the average coordination number increases, which is also reflected by the reorientational relaxation times in Fig. 3 in the main text. Again, both structural changes confirm the increasing red-shift of the O−H stretch as a function of increasing density with respect to the structural intermolecular distance-angle correlations which are well known from the literature [51–54]. The slight increase of the red-shift in SCW therefore perfectly correlates with the (only) slightly increasing average contribution of quasi-linear $O{-}H\cdots O$ angles between nearest-neighboring water molecules from ≈ 30 to $40\,\%$ upon increasing the density of SCW from 0.1 to $1.1\,\mathrm{kg\,L^{-1}}$ (again implying that the vast majority of water-water contacts features a strongly bent, non-linear such orientation). In conclusion, the small yet non-zero red-shift of the O−H stretching band in SCW does not imply the presence of H-bonding in SCW, especially because the red-shift is still too insignificant (see above), but merely reflects the much more pronounced thermal fluctuations in terms of significantly reduced H-bond lifetimes

and enhanced reorientational relaxation times which dramatically affect the intermolecular distance and angle distributions in SCW compared to RTW in the way just discussed.

III. STRUCTURAL ANALYSES

A. Joint Distribution Functions

In Fig. S7 we present joint angle-distance probability distribution functions (JDFs) of RTW and three representative supercritical state points at 750 K and densities of 0.1, 0.6, and $1.0\,\mathrm{kg\,L^{-1}}$. Additionally we depict in the insets the corresponding usual O−H radial distribution function (RDF) which can be obtained by integrating over the angle of the respective JDF. In case of RTW, see Fig. S7 (a), we observe that the H-bond peak around $r_{\mathrm{OH}} = 1.8\,\text{Å}$ and $\cos\Theta = -1$ (thus representing the ideal linear donor-acceptor H-bond orientation where $\Theta = 180°$) is well separated from the non H-bonded contributions implying a clear angular preference for a linear H-bond arrangement. This angular preference is also reflected by the corresponding RDF, where the H-bonded H atom is also well separated from all non H-bonded H atoms. When increasing the temperature this picture dramatically changes and the well-separated H-bonding peak in RTW smears out to larger distances and angles as visualized by the JDFs. As such the angular preference systematically diminishes as a function of temperature and further declines as a function of decreasing density until there is almost no preferential angular arrangement left at 750 K and $0.1\,\mathrm{kg\,L^{-1}}$ at all. It follows that the shoulder which appears in the O−H RDFs of SCW appears because the preference for linear H-bond arrangements in SCW is drastically reduced. It is worth to stress that the both, the relative height and shape of that shoulder with reference to the following first maximum of $g_{\mathrm{OH}}(r)$ is extremely similar at all supercritical densities ranging from 1.0 to $0.1\,\mathrm{kg\,L^{-1}}$.

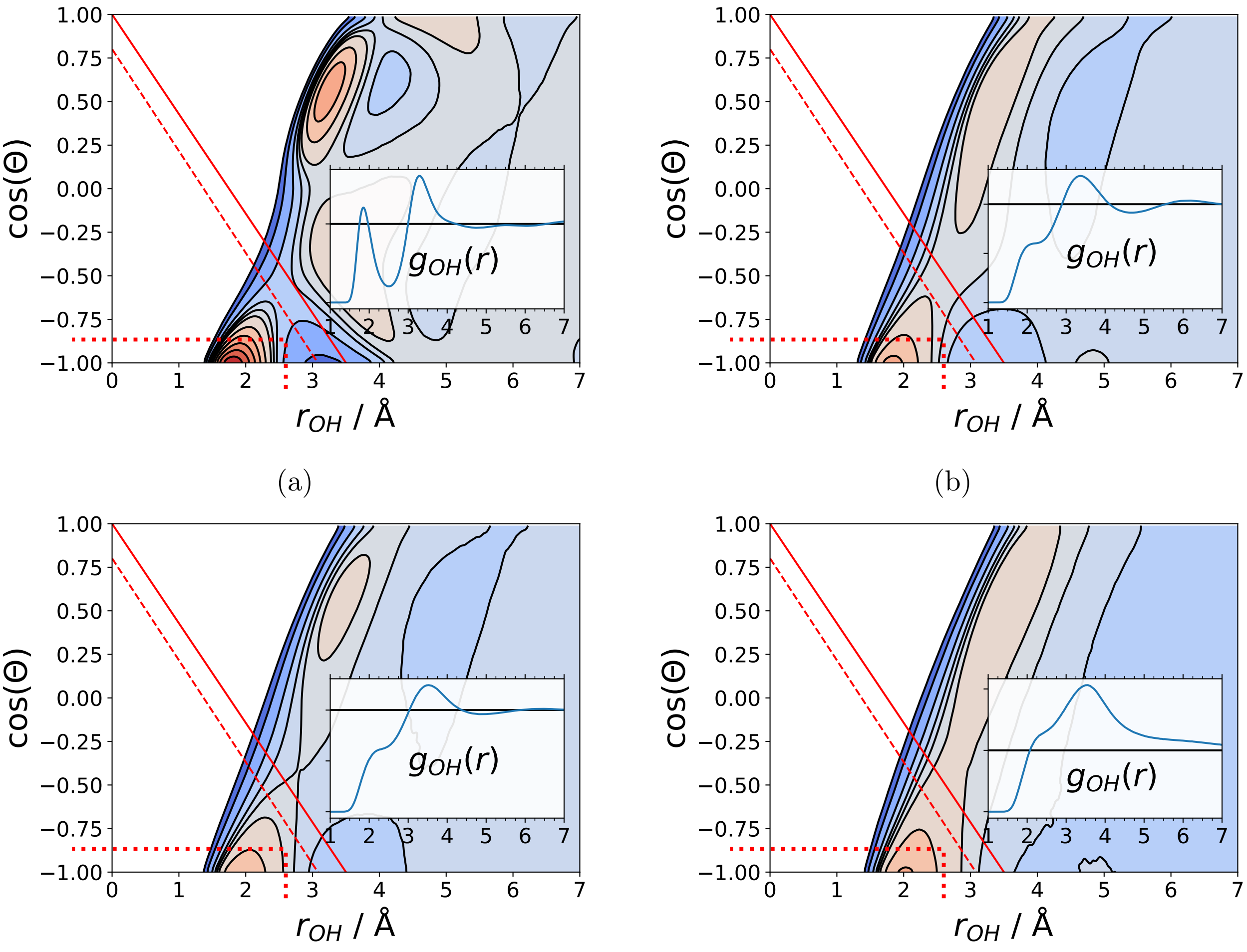

(a) (b)

(c) (d)

FIG. S7: Joint probability distribution functions of the structural arrangements of water pairs that describe the correlations between their intermolecular O · .. H distance r_{OH} and the corresponding intermolecular O−H · .. O angle Θ in case of liquid ambient water RTW (a) and supercritical water SCW at 750 K and densities of 1.0 (b), 0.6 (c), and 0.1 kg L^{-1} (d) of RPBE-D3 water. The probability increases from dark blue to dark red colors while white corresponds to zero probability. The contours are shown on a logarithmic scale and the same contour levels are used in all panels to allow for one-to-one comparison. The so-called SCW and RTW H-bond criteria, see text, according to Eq. (S9) are marked using red solid and dashed lines, respectively. Note that an angle of 180° ($\cos\Theta = -1$) corresponds to a perfectly linear O−H · .. O arrangement. The much used criterion according to Ref. 58 (wherein a pair of water molecules is considered to be H-bonded if $r_{\mathrm{HO}} < 2.6$ Å and $\Theta > 150°$) is included for reference using dotted red lines. The insets show the corresponding standard O−H radial distribution functions which can also be obtained by integrating over the angle Θ. The horizontal black lines indicate the reference value of $g_{\mathrm{OH}}(r) = 1$.

B. Hydrogen-Bond Criteria

To compute the continuous H-bond lifetime [59], we employ a joint angle-distance H-bond criterion

$$r_{\mathrm{HO}} < c_1^{\mathrm{HO}} + c_2^{\mathrm{HO}} \cos\Theta \ , \tag{S9}$$

where r_{HO} is the intermolecular $\mathrm{O}\cdots\mathrm{H}$ distance and Θ is the corresponding $\mathrm{O}\cdots\mathrm{H{-}O}$ angle. In case of ambient water, RTW, values of $c_1^{\mathrm{HO}} = 1.37\,\text{Å}$ and $c_2^{\mathrm{HO}} = -1.71\,\text{Å}$ have been shown to be useful [3, 11, 45]. However, in case of SCW a more lenient criterion has been applied [3, 4], namely $c_1^{\mathrm{HO}} = 1.75\,\text{Å}$ and $c_2^{\mathrm{HO}} = -1.75\,\text{Å}$, which accounts for the increased average $\mathrm{O}\cdots\mathrm{O}$ distances at supercritical conditions. These two criteria are dubbed "RTW" and "SCW" H-bond criterion, respectively, and are both illustrated in Fig. S7 together with the joint angle-distance distribution function that takes the relative arrangement of water pairs into account. Clearly, the RTW criterion (red dashed line) nicely separates the prominent peak in the lower-left corner due to H-bonded water pairs (at about 1.9 Å and $\cos\Theta = -1$) from non H-bonded structures in case of RTW, see panel (a) but it appears to be too strict in case of SCW even at the same high density according to panel (b). In contrast, the SCW criterion (red solid lines) clearly is too lenient to faithfully distinguish H-bonded from non H-bonded structures in case of RTW. We quantitatively assessed the effect of the chosen H-bond criterion on the H-bond lifetime by comparing in the main text the results obtained by the two distinct H-bond criteria. This direct comparison demonstrated that all conclusions drawn therein are robust with respect to that rather drastic change of the H-bond criterion.

IV. DETAILED COMPARISON BETWEEN OUR CONCLUSIONS AND AVAILABLE LITERATURE VIEWPOINTS

How does our conclusion of SCW not being H-bonded compare to existing viewpoints regarding its H-bonding properties? Let us start our discussion with ND/XRD experiments which yield the fully orientationally-averaged radial distance information, meaning RDFs, in the first place. Most interesting is the O−H RDF which features two distinct peaks at ambient conditions, RTW, where the first one can unambiguously be assigned to the intermolecular $\mathrm{O}\cdots\mathrm{H}$ distance of a H-bond. This situation becomes blurred at supercritical

conditions because the first peak vanishes in favor of an insignificant shoulder at sufficiently high temperatures as known since decades from e.g. Refs. 60–62. The appearance of that shoulder caused a vivid debate in the literature if H-bonds exist in SCW or not [60–66] with the current consensus [67] that at least some H-bonding persists in SCW, but it is significantly weakened. The results of our dynamical analysis of "orientationally preferred intermolecular encounters" (or H-bonding) in SCW as reported in the main text does not imply that there are no structural H-bond motives at all. But it certainly quantifies that they are remarkably short-lived, meaning that the barrier to break a H-bond is on average overcome due to thermal activation even before a single intermolecular ("H-bond") vibration can be exerted by a given water pair. This is exactly reflected in the shape of the corresponding RDFs in SCW which are found to systematically smear out as a function of increasing temperature and decreasing density. Importantly, since any RDF can be directly linked to the potential of mean force [68] we observe for the O−H RDFs (see insets Fig. S7) that there is no longer a local minimum and potential well separating H-bonded from non H-bonded structures in SCW, being in stark contrast to RTW.

In addition, since RDFs are time-averaged properties, they indeed capture even all transient orientational arrangements that formally correspond to orientational motives corresponding to linear H-bond arrangements irrespective of their possibly ultra-short lifetimes. In previous publications we have already shown that our ab initio simulations reproduce the experimental RDFs at comparable SCW state points reasonably well [3, 4], i.e. our simulations are indeed capable to closely reproduce these highly (time- and spatially-) averaged experimental observables. In Fig. S7 we present a correlation analysis of the joint angle-distance distribution functions of various supercritical state points in comparison to RTW. From these two-dimensional distribution functions one can immediately recognize that the preference for a linear H-bond arrangement is very significantly reduced in SCW even at high densities (b, c) compared to RTW (a) and it completely vanishes at low density (d). Note that it is possible to generate the corresponding O−H RDFs by explicitly integrating out the orientational dependence (implying averaging over $\cos\Theta$): This makes it absolutely evident that the transformation of the prominent first g_{OH} peak due to H-bonding in RTW (a) into a shoulder in SCW (b, c, d) occurs because the orientational preference – being a necessary ingredient of directional H-bonding – is lost. This implies that our conclusion from the main text of SCW not being H-bonded does not contradict the orientationally and time-averaged

structural information as provided by the RDFs stemming from ND/XRD experiments and computer simulations. To the contrary, it is quantitatively supported by those as demonstrated by Fig. S7.

Next, vibrational spectroscopy at mid-IR frequencies, see e.g. Refs 48–50, exclusively probes the stiff intramolecular O−H stretching motion of individual water molecules, and thus only provides indirect access to the intermolecular H-bond stretching dynamics due to water pairs. In RTW [69], the intramolecular O−H stretch is centered around $3500\,\mathrm{cm}^{-1}$ which corresponds to an oscillation period of about 10 fs. This spectral response blue shifts as a function of increasing temperature and decreasing density [48], i.e. the oscillation becomes even faster at supercritical conditions as is well understood. This implies that the intramolecular O−H stretch is more than one order of magnitude faster than the soft intermolecular O $\cdot$.. O stretching motion due to H-bonding as seen in the far-IR (THz) frequency regime. Thus, mid-IR methods are "fast" in this sense and thereby also sensitive to only fleetingly existing H-bonding contacts including those in SCW. Recall that we elaborately discuss in Sec. II D the (much reduced) red-shift of the intramolecular O−H stretch in SCW and indeed directly relate it to these short-lived H-bond contacts. Moreover, we show therein that this much reduced red-shift of the intramolecular O−H stretching band is due to the predominance of strongly bent, non-linear O−H $\cdot$.. O intermolecular angles in SCW, which do not qualify to be called H-bonds according to widespread use of the term in the context of bulk water [58]. As such, the mid-IR experiments substantially support our findings since the *intramolecular* O−H stretching vibrations are fast enough to probe and thus detect any instantaneous H-bond contacts even though these contacts are existing too shortly to be recognized by the *intermolecular* O $\cdot$.. O stretching vibrations in addition to also probing very non-linear water-water orientations which would not be called H-bonded according to established notions.

Finally, H-bonds in SCW have also been investigated using time-averaged chemical shift analysis of water molecules in ^{1}H NMR spectra [70] (as opposed to NMR relaxometry [71]). Here, it has been concluded that H-bonding in SCW is reduced to about 29 % of that in liquid water. However, the authors themselves explicitly spell out that the measured chemical shift reflects a time average over about 0.1 s. Generally, NMR is known as a "slow method", i.e. a single number is extracted as a time-average of a multitude of fluctuating intermolecular arrangements within the fluid. Therefore, analyzing the chemical shift suffers

from the very same problem as already discussed for the RDFs, namely that all instantaneous H-bond contacts are detected although they are only fleetingly existing on the timescale of the relevant intermolecular $O \cdot .. O$ vibrations as indeed supported by the time-resolved NMR relaxometry data [71] presented in Fig. 3 (b).

We conclude that our data and our interpretation are consistent with available experimental and computational literature data. Yet, THz spectra offer a complementary view which extends the insights based on time- and orientationally-averaged data as provided by ^{1}H NMR, ND or XRD as well as from fast mid-IR spectroscopy. Intermolecular encounters in linear H-bond arrangements are therefore existing for too short times in SCW to be recognized as intermolecular water–water vibrations, but they still live long enough to be recognized by the fast intramolecular O−H stretch and are also seen in time- and spatially-averaged observables. In any case, the question remains if it is still useful to call a fluid H-bonded if the required directional linear orientations of two molecules do not survive even a single oscillation period within that linear "anisotropic two-body arrangement" that is usually called a H-bond.

[1] D. Marx and J. Hutter, *Ab Initio Molecular Dynamics: Basic Theory and Advanced Methods* (Cambridge University Press, Cambridge, 2009).

[2] P. Schienbein and D. Marx, Phys. Rev. E **98**, 022104 (2018).

[3] P. Schienbein and D. Marx, J. Phys. Chem. B **122**, 3318 (2018).

[4] P. Schienbein and D. Marx, Phys. Chem. Chem. Phys. , DOI: 10.1039/C9CP05610F (2020).

[5] M. Heyden, J. Sun, S. Funkner, G. Mathias, H. Forbert, M. Havenith, and D. Marx, Proc. Natl. Acad. Sci. U.S.A. **107**, 12068 (2010).

[6] M. Heyden, J. Sun, H. Forbert, G. Mathias, M. Havenith, and D. Marx, J. Phys. Chem. Lett. **3**, 2135 (2012).

[7] V. P. Sokhan, A. Jones, F. Cipcigan, J. Crain, and G. J. Martyna, Phys. Rev. Lett. **115**, 117801 (2015).

[8] K. Tonigold and A. Groß, J. Comput. Chem. **33**, 695 (2012).

[9] T. Morawietz and J. Behler, J. Phys. Chem. A **117**, 7356 (2013).

[10] K. Forster-Tonigold and A. Groß, J. Chem. Phys. **141**, 064501 (2014).

[11] S. Imoto, H. Forbert, and D. Marx, Phys. Chem. Chem. Phys. **17**, 24224 (2015).

[12] S. Sakong, K. Forster-Tonigold, and A. Groß, J. Chem. Phys. **144**, 194701 (2016).

[13] T. Morawietz, A. Singraber, C. Dellago, and J. Behler, Proc. Natl. Acad. Sci. U.S.A. **113**, 8368 (2016).

[14] S. Imoto, P. Kibies, C. Rosin, R. Winter, S. M. Kast, and D. Marx, Angew. Chem. Int. Ed. **55**, 9534 (2016).

[15] H. Vondracek, S. Imoto, L. Knake, G. Schwaab, D. Marx, and M. Havenith, J. Phys. Chem. B **123**, 7748 (2019).

[16] O. Marsalek and T. E. Markland, J. Phys. Chem. Lett. **8**, 1545 (2017).

[17] J. VandeVondele, F. Mohamed, M. Krack, J. Hutter, M. Sprik, and M. Parrinello, J. Chem. Phys. **122**, 014515 (2005).

[18] J. Hutter, M. Iannuzzi, F. Schiffmann, and J. VandeVondele, WIREs Comput. Mol. Sci. **4**, 15 (2014).

[19] J. P. Perdew, K. Burke, and M. Ernzerhof, Phys. Rev. Lett. **77**, 3865 (1996), Erratum: Phys. Rev. Lett. **78**, 1396–1396 (1997).

[20] B. Hammer, L. B. Hansen, and J. K. Nørskov, Phys. Rev. B **59**, 7413 (1999).

[21] Y. Zhang and W. Yang, Phys. Rev. Lett. **80**, 890 (1998).

[22] M. A. Marques, M. J. Oliveira, and T. Burnus, Comput. Phys. Commun. **183**, 2272 (2012).

[23] S. Grimme, J. Antony, S. Ehrlich, and H. Krieg, J. Chem. Phys. **132**, 154104 (2010).

[24] S. Goedecker, M. Teter, and J. Hutter, Phys. Rev. B **54**, 1703 (1996).

[25] C. Hartwigsen, S. Goedecker, and J. Hutter, Phys. Rev. B **58**, 3641 (1998).

[26] G. Lippert, J. Hutter, and M. Parrinello, Mol. Phys. **92**, 477 (1997).

[27] J. VandeVondele and J. Hutter, J. Chem. Phys. **127**, 114105 (2007).

[28] M. Guidon, J. Hutter, and J. VandeVondele, J. Chem. Theory Comput. **6**, 2348 (2010).

[29] A. Bankura, A. Karmakar, V. Carnevale, A. Chandra, and M. L. Klein, J. Phys. Chem. C **118**, 29401 (2014).

[30] R. C. Remsing, M. D. Baer, G. K. Schenter, C. J. Mundy, and J. D. Weeks, J. Phys. Chem. Lett. **5**, 2767 (2014).

[31] L. B. Skinner, M. Galib, J. L. Fulton, C. J. Mundy, J. B. Parise, V.-T. Pham, G. K. Schenter, and C. J. Benmore, J. Chem. Phys. **144**, 134504 (2016).

[32] W. Wagner and A. Pruß, J. Phys. Chem. Ref. Data **31**, 387 (2002).

[33] J.-P. Hansen and L. Verlet, Phys. Rev. **184**, 151 (1969).

[34] J. J. Potoff and A. Z. Panagiotopoulos, J. Chem. Phys. **109**, 10914 (1998).

[35] J. S. Rowlinson, *Liquids and Liquid Mixtures, 2nd Edition* (Butterworth, London, 1969).

[36] R. Ramírez, T. López-Ciudad, P. P. Kumar, and D. Marx, J. Chem. Phys. **121**, 3973 (2004).

[37] IUPAC, "Compendium of chemical terminology, 2nd ed. (the "gold book")," (1997).

[38] R. Iftimie and M. E. Tuckerman, J. Chem. Phys. **122**, 214508 (2005).

[39] M. Śmiechowski, J. Sun, H. Forbert, and D. Marx, Phys. Chem. Chem. Phys. **17**, 8323 (2015).

[40] A. H. Harvey, J. S. Gallagher, and J. M. H. L. Sengers, J. Phys. Chem. Ref. Data **27**, 761 (1998).

[41] *Release on the Refractive Index of Ordinary Water Substance as a Function of Wavelength, Temperature and Pressure* (The International Association for the Properties of Water and Steam, Erlangen, Germany, 1997. http://iapws.org/relguide/rindex.pdf).

[42] W. H. Press, S. A. Teukolsky, W. T. Vetterling, and B. P. Flannery, *Numerical Recipies – Third Edition* (Cambridge University Press, Cambridge, 2007).

[43] J. Martí, Phys. Rev. E **61**, 449 (2000).

[44] S. Imoto and D. Marx, J. Chem. Phys. **150**, 084502 (2019).
[45] P. Schienbein, G. Schwaab, H. Forbert, M. Havenith, and D. Marx, J. Phys. Chem. Lett. **8**, 2373 (2017).
[46] A. Esser, H. Forbert, F. Sebastiani, G. Schwaab, M. Havenith, and D. Marx, J. Phys. Chem. B **122**, 1453 (2018).
[47] P. K. Gupta, P. Schienbein, J. Daru, and D. Marx, J. Phys. Chem. Lett. **10**, 393 (2019).
[48] E. U. Franck and K. Roth, Discuss. Faraday Soc. **43**, 108 (1967).
[49] J. D. Frantz, J. Dubessy, and B. Mysen, Chem. Geol. **106**, 9 (1993).
[50] N. J. Hestand, S. E. Strong, L. Shi, and J. L. Skinner, J. Chem. Phys. **150**, 054505 (2019).
[51] E. Libowitzky, Chem. Monthly **130**, 1047 (1999).
[52] R. Rey, K. B. Møller, and J. T. Hynes, J. Phys. Chem. A **106**, 11993 (2002).
[53] C. P. Lawrence and J. L. Skinner, J. Chem. Phys. **118**, 264 (2003).
[54] C. J. Fecko, J. D. Eaves, J. J. Loparo, A. Tokmakoff, and P. L. Geissler, Science **301**, 1698 (2003).
[55] S. A. Corcelli and J. L. Skinner, J. Phys. Chem. A **109**, 6154 (2005).
[56] J. J. Loparo, S. T. Roberts, R. A. Nicodemus, and A. Tokmakoff, Chem. Phys. **341**, 218 (2007).
[57] S. D. Ivanov, A. Witt, and D. Marx, Phys. Chem. Chem. Phys. **15**, 10270 (2013).
[58] A. Luzar and D. Chandler, Nature **379**, 55 (1996).
[59] D. C. Rapaport, Mol. Phys. **50**, 1151 (1983).
[60] P. Postorino, R. H. Tromp, M. A. Ricci, A. K. Soper, and G. W. Neilson, Nature **366**, 668 (1993).
[61] R. H. Tromp, P. Postorino, G. W. Neilson, M. A. Ricci, and A. K. Soper, J. Chem. Phys. **101**, 6210 (1994).
[62] A. K. Soper, F. Bruni, and M. A. Ricci, J. Chem. Phys. **106**, 247 (1997).
[63] A. K. Soper, J. Phys.: Condens. Matter **8**, 9263 (1996).
[64] M.-C. Bellissent-Funel, T. Tassaing, H. Zhao, D. Beysens, B. Guillot, and Y. Guissani, J. Chem. Phys. **107**, 2942 (1997).
[65] T. Tassaing, M.-C. Bellissent-Funel, B. Guillot, and Y. Guissani, Europhys. Lett. **42**, 265 (1998).
[66] A. Botti, F. Bruni, M. A. Ricci, and A. K. Soper, J. Chem. Phys. **109**, 3180 (1998).

[67] Y. Marcus, *Supercritical Water* (John Wiley & Sons, Inc., Hoboken, New Jersey, 2012).

[68] D. Chandler, *Introduction to Modern Statistical Mechanics* (Oxford University Press, Oxford, New York, Toronto, 1987).

[69] J. E. Bertie and Z. Lan, Appl. Spectrosc. **50**, 1047 (1996).

[70] M. M. Hoffmann and M. S. Conradi, J. Am. Chem. Soc. **119**, 3811 (1997).

[71] N. Matubayasi, N. Nakao, and M. Nakahara, J. Chem. Phys. **114**, 4107 (2001).
